# Predictable Self‐Assembly as an Unexplored Key Factor Influencing Membrane Separation: Insights from Monophenols

**DOI:** 10.1002/advs.202504322

**Published:** 2025-06-20

**Authors:** Qiuyu Han, Lu Yin, Tingting Mi, Qi Chen, Wanlin Ouyang, Liping Fan, Qinshi Wang, Yue Zhang, Zhishu Tang, Huaxu Zhu, Bo Li

**Affiliations:** ^1^ Jiangsu Collaborative Innovation Center of Chinese Medicinal Resources Industrialization Nanjing University of Chinese Medicine Nanjing 210023 China; ^2^ Jiangsu Research Center of Botanical Medicine Refinement Engineering Nanjing University of Chinese Medicine Nanjing 210023 China; ^3^ The First Clinical Medical College Nanjing University of Chinese Medicine Nanjing 210023 China; ^4^ School of Chinese Materia Medica Beijing University of Chinese Medicine Beijing 100029 China

**Keywords:** π‐π stacking interaction, monophenols, nanofiltration, self‐assembly, separation

## Abstract

While nanofiltration (NF) holds promise for separating small molecules, effectively separating structurally similar compounds like monophenols remains challenging. This study unveils a novel NF separation strategy based on the often‐overlooked phenomenon of solute self‐assembly. Using a combination of experimental and computational approaches, a direct link between monophenol self‐assembly and rejection behavior during NF is established. The self‐assembly of monophenols, primarily driven by π–π stacking interactions, is shown to significantly influence their rejection rates, with larger, more numerous self‐assemblies experiencing higher rejection. Furthermore, a clear relationship between monophenol structures and self‐assembly strength is established, revealing that the number and Hydrogen (H)‐bonding capacity of substituents on the aromatic ring dictate the propensity for self‐assembly. This insight enables the development of a predictive model for monophenol self‐assembly, which is validated through NF experiments using binary mixtures, confirming that predictable differences in self‐assembly behavior can be leveraged for selective separation. This study establishes solute self‐assembly as a tunable parameter for enhancing NF separation of similarly sized molecules.

## Graphical abstract

Predictable molecular self‐assembly strength is key to membrane separation, contrary to typical understanding. Monophenol research shows substituent abundance and specificity govern self‐assembly strength. This predictability enables a novel separation method: membranes reject molecules with stronger self‐assembly more effectively, separating similarly sized molecules. This finding transcends conventional nanofiltration relying solely on molecular size and charge for separation.

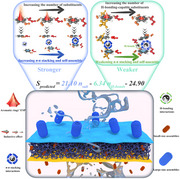

## 1 Introduction

Monophenols, derived from the shikimic acid and phenylpropanoid secondary metabolic pathways,^[^ ^1^ ^]^ are an important class of bioactive compounds found in plants. They exhibit antioxidant, anti‐inflammatory, antibacterial, and antithrombotic effects, with significant applications in the treatment of cancer and diabetes.^[^ ^2^, ^3^, ^4^, ^5^, ^6^ ^]^ Monophenols are essential pharmaceutical compounds with diverse therapeutic benefits. However, their efficacy depends on their chemical structure.^[^ ^7^, ^8^, ^9^, ^10^, ^11^ ^]^ Thus, separating, purifying, and concentrating monophenols are crucial, regardless of whether they are synthesized or extracted from plants. Traditional methods for effective monophenols separation have been phased out in industrial applications, due to low yields, complex procedures, high energy consumption, and environmental risks.^[^ ^12^, ^13^, ^14^, ^15^, ^16^ ^]^ Consequently, developing new and improved technologies is critical.

Membrane separation technology is gaining traction as a replacement for traditional separation methods due to its superior separation efficiency, low energy consumption, and environmental benefits.^[^ ^17^, ^18^ ^]^ Nanofiltration (NF) is particularly well‐suited for separating small molecules.^[^ ^19^, ^20^, ^21^, ^22^, ^23^, ^24^ ^]^ NF separates substances by exploiting differences in their physicochemical properties. While previous studies exploring these differences have primarily focused on Stokes radius, *pKa*, and *logD* to explain rejection behavior,^[^ ^25^ ^]^ the role of solute self‐assembly is often overlooked. Stokes radius governs size exclusion effects,^[^ ^26^, ^27^, ^28^, ^29^ ^]^ while *pKa* and *logD* influence a solute's affinity for the membrane.^[^ ^30^, ^31^, ^32^, ^33^, ^34^, ^35^ ^]^ However, the formation of supramolecular aggregates through self‐assembly can lead to unexpected rejection behavior. For instance, Jiang et al.^[^ ^36^ ^]^ observed unexpectedly high rejection rates for dyes during ultrafiltration, attributing this to dye cluster formation. Similarly, our previous work shows that the self‐assembly of salvianolic acid B significantly influences its rejection behavior during the membrane process.^[^ ^37^ ^]^ These findings demonstrate that considering solute self‐assembly is crucial for understanding rejection behavior, highlighting its significance as a key mechanism in membrane separations.

Self‐assembly, the spontaneous organization of components into ordered structures without external intervention,^[^ ^38^ ^]^ is a ubiquitous phenomenon in nature. From DNA and proteins to crystals and colloids, numerous substances form through molecular self‐assembly driven by weak interactions.^[^ ^39^, ^40^, ^41^, ^42^ ^]^ These interactions include electrostatic, van der Waals, hydrophobic, Hydrogen (H)‐bonding, and π‐π stacking interactions.^[^ ^43^, ^44^, ^45^, ^46^ ^]^ The fundamental principles governing these interactions enable the prediction and control of self‐assembly, leading to the development of self‐assembly technologies.^[^ ^47^ ^]^ The technologies have been widely applied in surfactants,^[^ ^48^, ^49^ ^]^ pharmacology,^[^ ^50^, ^51^, ^52^, ^53^, ^54^, ^55^ ^]^ materials science.^[^ ^56^, ^57^, ^58^, ^59^ ^]^ Importantly, many molecules exist as nanoscale self‐assemblies rather than in free form before forming more complex structures.^[^ ^60^, ^61^, ^62^, ^63^, ^64^ ^]^ These self‐assemblies, observed in numerous small molecule clusters,^[^ ^65^, ^66^, ^67^ ^]^ can significantly impact membrane processes, potentially increasing rejection or fouling.

Two primary mechanisms can lead to the self‐assembly of monophenols and their analogs. One involves complexation with multivalent metal cations,^[^ ^68^, ^69^, ^70^ ^]^ while the other relies on self‐assembly driven by weak interactions.^[^ ^71^ ^]^ This latter mechanism typically results in predominantly columnar structures, as observed in gallic acid (GA), caffeic acid (CA), and dopamine.^[^ ^72^, ^73^, ^74^ ^]^ Self‐assembly of monophenols, often overlooked, can cause abnormal rejection in membrane processes. Therefore, a deeper understanding of the mechanisms governing monophenol self‐assembly and its effect on membrane processes is needed. This understanding may also address a key question: Can variations in self‐assembly be exploited for selective separation? Monophenols served as the focus of this study.

This study tackled this challenge by establishing predictable solute self‐assembly as a key factor influencing rejection behavior, thus providing a novel strategy for the membrane separation of different monophenols. The critical role of monophenol self‐assembly in governing rejection behavior during NF was first elucidated using macroscopic membrane rejection experiments. These findings were further investigated with microscopic molecular dynamics simulations (MDS). Atomic‐level quantum chemical (QC) analyses were then employed to establish a systematic, predictable model linking monophenol structures to their self‐assembly strengths. The pattern was precisely validated through membrane separation experiments involving binary monophenols mixtures. This study significantly refined current understanding of membrane separation mechanisms and paved the way for more efficient and targeted separations by leveraging solute self‐assembly.

## 2 Results and Discussion

### 2.1 Analysis of Monophenols Rejection in Unary‐Systems

#### 2.1.1 Analysis of Mass Transfer Behavior

**Figure 1:**
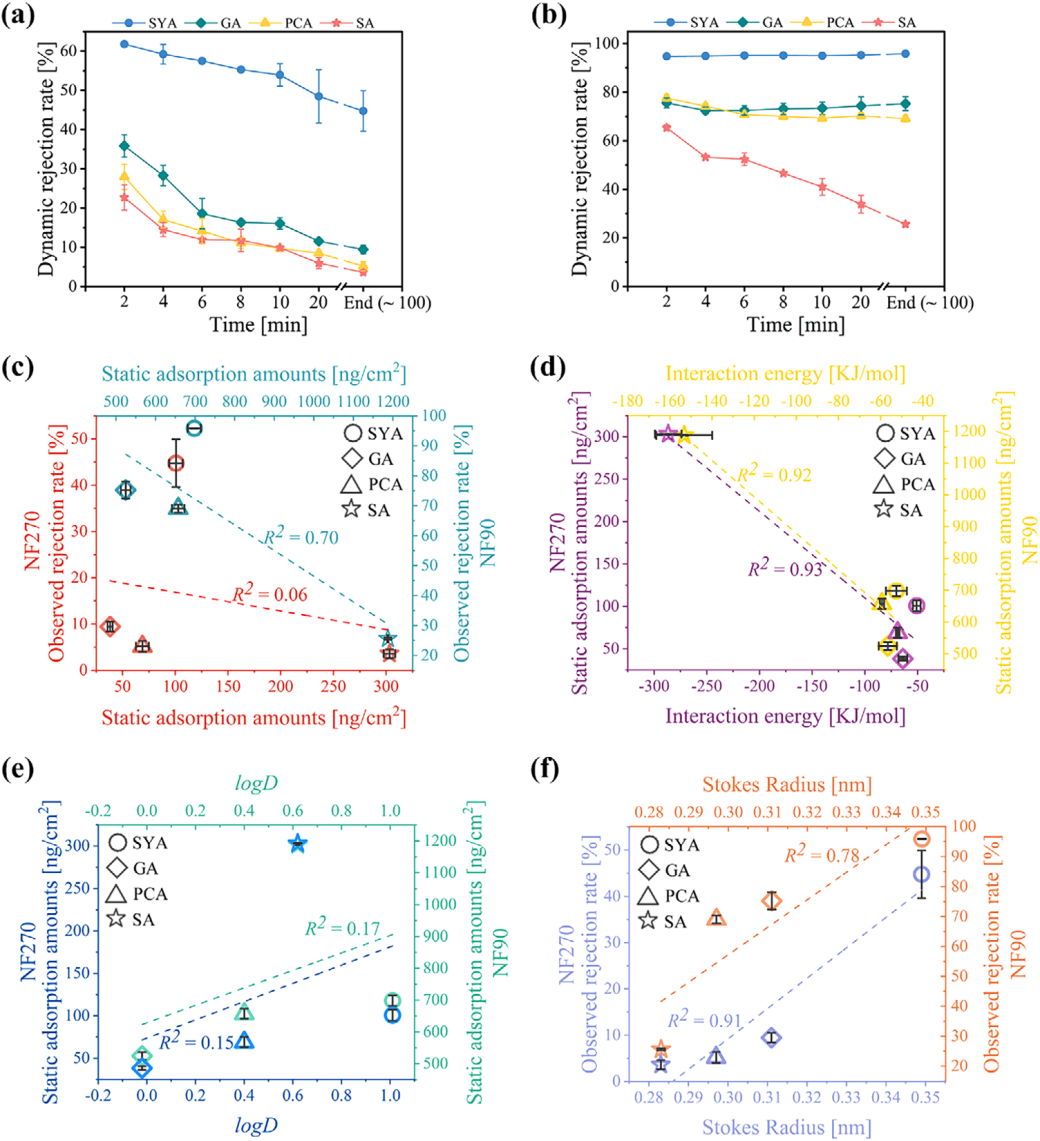
Analysis of the mass transfer behavior for monophenols. a) Dynamic rejection rates in the NF270. b) Dynamic rejection rates in the NF90. c) Correlations between the static adsorption amounts and observed rejection rates. d) Correlations between the static adsorption amounts and interaction energies (electrostatic plus van der Waals). e) Correlations between the static adsorption amounts and hydrophobic interactions (Using monophenols’ *logD* as an indicator). f) Correlation between the Stokes radius and observed rejection rates.

This study examined the rejection behavior of monophenols at their original *pH*. The dynamic rejection rate of most monophenols gradually decreased during NF (**Figure** **1**a,b). Notably, syringic acid (SYA), GA, protocatechuic acid (PCA), and salicylic acid (SA) showed a sequential decline in rejection rates. This could be explained by the solution‐diffusion model.^[^ ^75^, ^76^ ^]^ Initially, high rejection was observed due to the opposing effects of adsorption and diffusion. As adsorption sites saturated, monophenols with stronger membrane affinity induced greater concentration polarization and solubility, leading to lower rejection. To validate this, the affinity of the membrane surfaces for monophenols has been investigated.

​The static adsorption amounts were utilized to quantify the affinity. However, Figure 1c reveals a weak correlation between the adsorption amounts of monophenols and their observed rejection rates, suggesting that affinity alone minimally affected rejection. This was because, at the original pH, the affinity was primarily governed by their interaction energies (van der Waals plus electrostatic) (Figure 1d,e). Given that both monophenols and membranes were nearly neutral at their original pH (**Table** **1**; Figure S1, Supporting Information), the overall interaction energies between them remained low.^[^ ^77^, ^78^ ^]^ This meant that affinity's contribution to rejection was minor, allowing other more influential factors to easily dominate. Molecular size was a stronger factor influencing monophenols rejection (Figure 1f). However, the impact of size varied significantly between membrane systems, acting as a dominant factor for NF270 but exhibiting a weaker influence on NF90. This indicates that other additional factors, beyond affinity and size, might be influencing monophenols rejection.

**Table 1:** Key physicochemical properties for monophenols.

Compound	SYA	GA	PCA	SA	FA	CA
Molecular formula	C_9_H_10_O_5_	C_7_H_6_O_5_	C_7_H_6_O_4_	C_7_H_6_O_3_	C_10_H_10_O_4_	C_9_H_8_O_4_
Structure	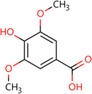	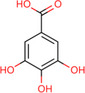	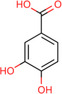	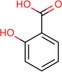	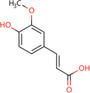	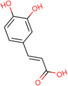
Mw [g mol⁻^1^]	198.17	170.12	154.12	138.12	194.18	180.16
pH	3.30	3.23	3.23	2.59	3.6	3.8
Stokes radius [nm] ^a)^	0.349	0.311	0.297	0.283	0.358	0.338
*pKa* ^b)^	3.93	3.94	4.16	2.79	3.97	3.84
Charge ^b)^	− 0.1892	− 0.1535	− 0.0988	− 0.3925	− 0.3003	− 0.4748
Solubility [mmol L⁻^1^] ^b)^	83.52	599.05	229.24	139.83	27.17	81.58
*LogP* ^c)^	1.10	0.06	0.45	0.83	1.70	1.18
*LogD* ^d)^	1.01	− 0.02	0.40	0.62	1.55	0.90

^a)^ The Data was evaluated using the Stokes‐Einstein equation, which assumed spherical solutes. The diffusivity utilized in the Stokes‐Einstein equation was determined by the Wilke‐Chang equation;

^b)^ Data under original pH calculated with ChemAxon (http://www.chemicalize.com);

^c)^ Data cited from the literatures^[^ ^81^ ^]^;

^d)^ Data calculated by the *logD* equation.^[^ ^82^ ^]^

Given the higher rejection rates of monophenols compared to similarly sized molecules ​with their rejection primarily governed by molecular size,^[^ ^79^, ^80^ ^]^ we hypothesized that varying‐sized supramolecular self‐assemblies formed by different monophenols might contribute to the observed rejection behavior. Corresponding experimental techniques were next adopted to characterize the monophenol self‐assemblies in solutions and membrane processes.

#### 2.1.2 Experimental Characterization of Monophenol Self‐Assemblies

**Figure 2:**
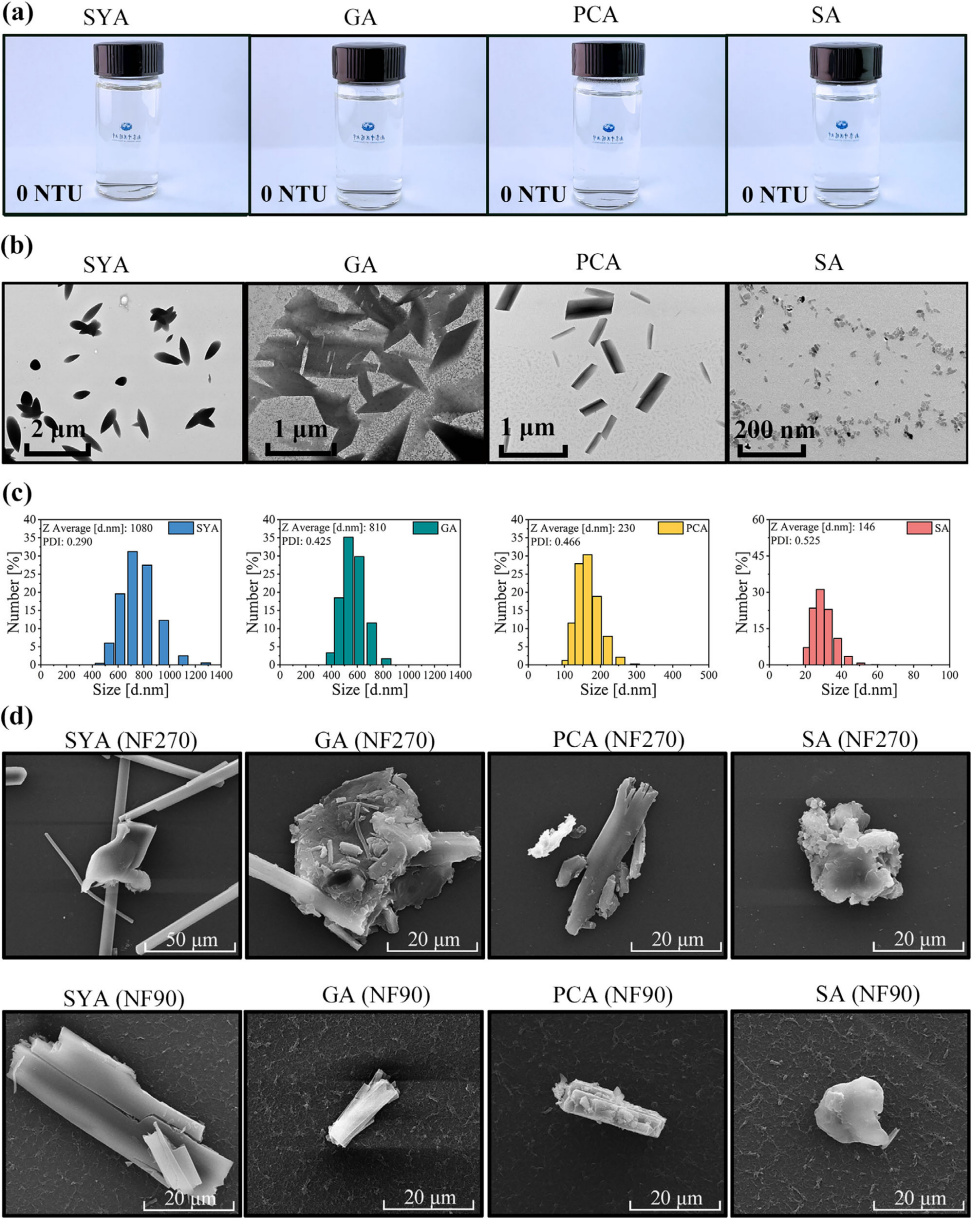
Experimental characterization of monophenol self‐assemblies. a) Four Feed solutions of monophenols in NF. b) Transmission Electron Microscopy (TEM) image of the feed solutions. c) Size distribution in the feed solutions. d) Scanning Electron Microscopy (SEM) images of membrane surfaces morphology after NF (The blank control of the membrane surfaces is shown in Figure S3, Supporting Information).

The four monophenol solutions used in the NF had a concentration of 10 mmol L⁻^1^ (below their solubility, Table 1), exhibiting clarity and transparency with a turbidity level of 0 NTU (**Figure** **2**a) and not observing the Tyndall effect (Figure S2, Supporting Information). Despite their clarity, the monophenols partially formed rod‐shaped nanoparticles rather than remaining fully free in solution (Figure 2b). Moreover, particle size distributions in the solutions followed a distinct order: SYA > GA > PCA > SA. (Figure 2c). This order reflects the relative strength of self‐assembly among the four monophenols and aligns with the observed rejection rates (Figure 1a,b), implying a causal relationship between self‐assembly and rejection. Specifically, monophenols with stronger self‐assembly effects tended to form larger, more numerous self‐assemblies and reduce free molecules, resulting in higher rejection rates.

Notably, self‐assemblies on the membrane surfaces after NF were consistently larger than those in the bulk solution (Figure 2d). This occurred because the concentration‐dependent self‐assembly effect became amplified under the high‐concentration conditions of the polarization layer inherent to membrane processes,^[^ ^83^, ^84^, ^85^, ^86^ ^]^ driving the formation of larger self‐assemblies. This underscores the necessity of considering solute's self‐assembly effects in membrane science. To further confirm the differences in the self‐assembly of various monophenols, an in‐depth investigation was conducted using MDS.

### 2.2 Analysis of Self‐Assembly Patterns for Monophenols

#### 2.2.1 Analysis of Differences in Self‐Assembly Effects

**Figure 3:**
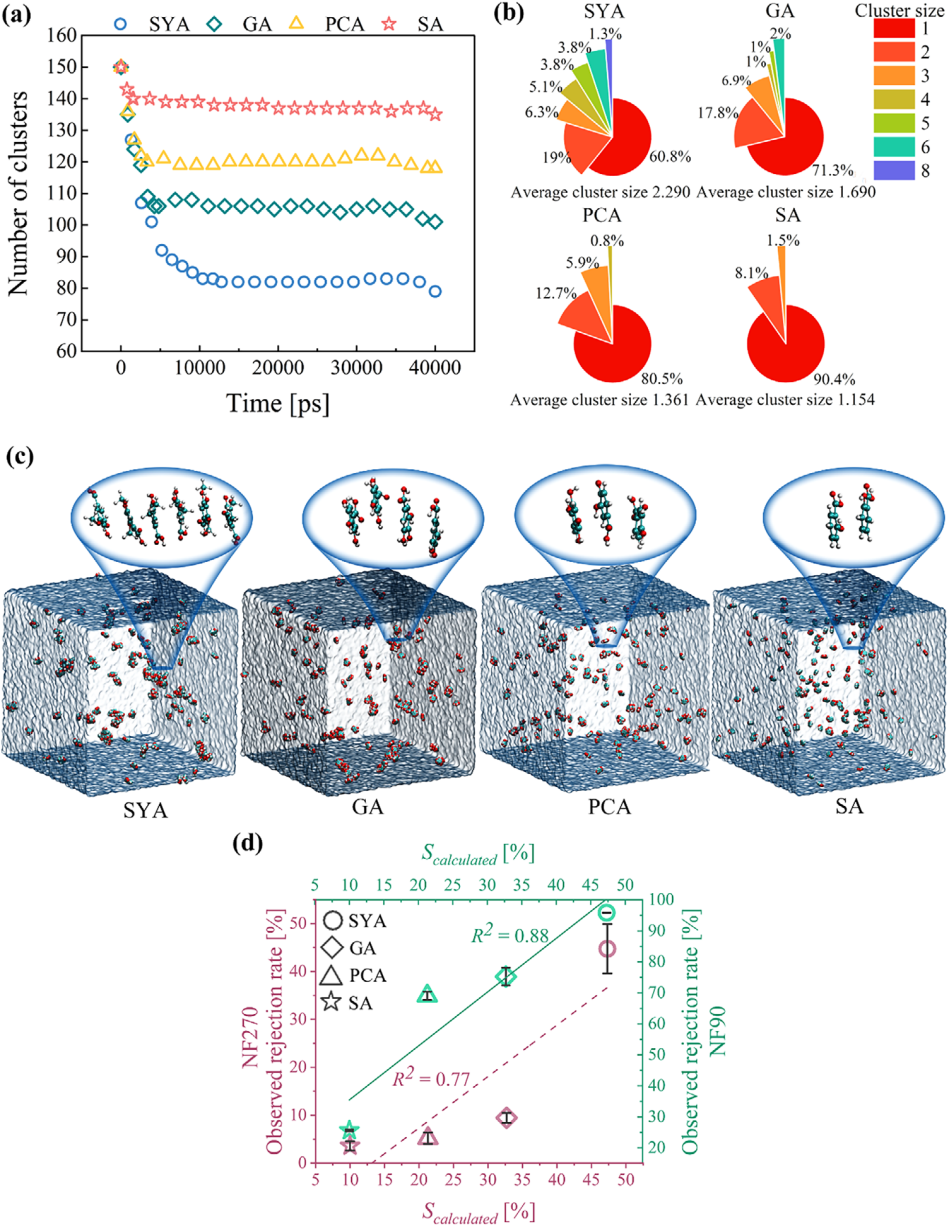
Analysis of Differences in Self‐Assembly Effects. a) Variation in the number of monophenol clusters over the 40 ns production runs. b) The distribution of monophenol clusters sizes after 40 ns production runs (cluster size was measured in molecular count; larger clusters contained more molecules.^[^ ^87^, ^88^ ^]^). c) Final snapshot of different monophenol clusters. d) Correlation between the *S_calculated_* and observed rejection rate of monophenols.

Analysis of 40 ns production runs reveals that the number of monophenol clusters decreased and plateaued within the first 10 ns for all systems (**Figure** **3**a). This indicates rapid self‐assembly of the monophenols throughout the membrane filtration process.

Further analysis of cluster sizes in the final frame reveals a distinct order for the size of free clusters among the four systems: SYA > GA > PCA > SA (Figure 3b,c). This order is in full agreement with Figure 2c, further confirming the regular differences in self‐assembly among the monophenols. To quantitatively explore the impact of monophenols’ self‐assembly on rejection, we introduced the concept of a self‐assembly rate (*S*) as an indicator of self‐assembly strength. It is calculated using the following equation:

(1) $$S_{calculated}\;\;\left(\%\right)=\frac{n_{initial}-n_{final}}{n_{initial}}\;*\;100$$

Where *S_calculated_* represented the self‐assembly rate computed from MDS data. *n_initial_* represented the number of free clusters in the initial system during the production runs (typically equal to the number of molecules introduced), and *n_final_* represented the number of free clusters in the final frame of the production runs.

The *S_calculated_* strongly correlates with the rejection rate, highlighting its significant influence on rejection (Figure 3d). Interestingly, the impact of self‐assembly was more pronounced for the NF90 membrane compared to the NF270 membrane. This difference was likely due to the concentration‐dependent nature of self‐assembly. The higher concentration within the NF90 membrane's polarization layer would promote more intense self‐assembly and, consequently, greater rejection.

In summary, this confirms that monophenols’ self‐assembly was another key factor influencing their rejection behavior. Monophenols with stronger self‐assembly effects tended to form larger and more numerous self‐assemblies, resulting in higher rejection rates. To further elucidate the underlying causes for these differences in self‐assembly behavior, an analysis of the driving forces behind monophenol self‐assembly was conducted.

#### 2.2.2 Analysis of Driving Forces for Monophenols’ Self‐Assembly

##### Analysis via MDS Methods

**Figure 4:**
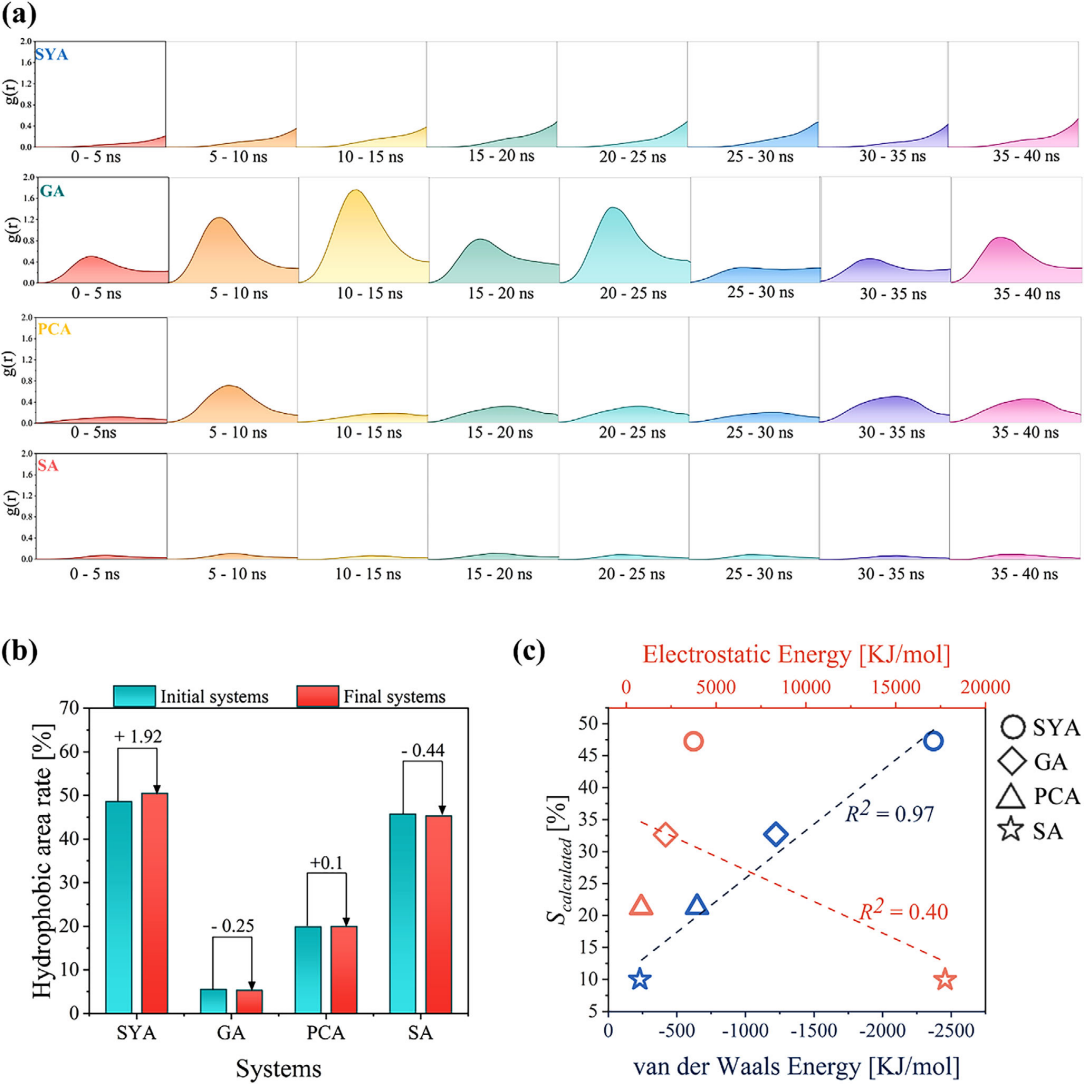
Analysis of driving forces for self‐assembly. a) The RDFs of intermolecular H‐bonds every 5 ns during production runs (excl = 3000, bin = 0.002, *r_min_* = 0.13, *r_max_* = 0.2). b) Hydrophobic area rate for both the initial and final systems in production runs. c) Correlation between the overall intermolecular electrostatic and van der Waals energies for various monophenols and their *S_calculated_*.

To assess the role of H‐bonding interactions in the self‐assembly process, intermolecular O – H radial distribution functions (RDFs) were analyzed every 5 ns during the production runs (The gmx‐hbond tool was excluded for its inability to distinguish intramolecular from intermolecular H‐bonds). Figure S4 (Supporting Information) shows RDF peaks for intermolecular H‐bonds within 0.13–0.2 nm,^[^ ^89^, ^90^, ^91^, ^92^ ^]^ further magnified in **Figure** **4**a. Notably, SYA and SA exhibited almost no intermolecular H‐bonds, while GA and PCA showed few and irregular fluctuations in their number. These results indicate that intermolecular H‐bonds between monophenols were infrequent, unstable, and not a driving force for self‐assembly.

To characterize the impact of hydrophobic interactions, the solvent‐accessible surface area and the rate of hydrophobic surface area (calculated by Equation S1, Supporting Information)^[^ ^93^, ^94^ ^]^ were analyzed. The hydrophobic surface area rate of monophenols remained nearly unchanged before and after self‐assembly (Figure 4b), indicating that hydrophobic interactions did not drive this process.^[^ ^95^, ^96^, ^97^ ^]^ And H‐bonds between solutes and water were critical for dissolution. However, Figure S5a,b (Supporting Information) shows that the self‐assembly of monophenols reduced the number of water molecules on their surfaces while maintaining stable H‐bonding counts between monophenols and water (except for GA). This confirms that the self‐assembly regions between monophenols were devoid of H‐bonds with water molecules, and H‐bonds between monophenols and water did not affect the self‐assembly (The minor reduction in GA−water H‐bonds arose from stronger intermolecular H‐bonds formation, displacing aqueous interactions, Figure S5c,d, Supporting Information). Collectively, these particular water‐mediated effects did not appear to be the primary factors driving the observed differences in self‐assembly among the monophenols.

The overall electrostatic and van der Waals energies between monophenols in different systems were analyzed during production runs. Figure 4c shows that electrostatic forces had no discernible impact on self‐assembly (Distinct from charge‐driven self‐assembly approaches, this study examined single‐constituent self‐assembly, a system composed of molecules with the same charge, in which charge interactions did not constitute the principal driving force.). In contrast, van der Waals forces showed a strong positive correlation with the *S_calculated_*. This finding, along with the observation of parallel‐displaced stacking of aromatic rings in typical self‐assemblies (Figure 3c), confirms that van der Waals forces arising from π–π stacking interactions were the primary driving force for monophenols’ self‐assembly. And the distinct redshift in UV–vis spectra of higher‐concentration monophenol solutions with more self‐assemblies, compared to lower‐concentration ones, also experimentally demonstrates π‐π stacking's critical role in driving self‐assembly (Figure S6, Supporting Information). To further investigate the observed differences in π–π stacking interactions among monophenols (SYA > GA > PCA > SA), QC methods with smaller analytical scale were employed.

##### Analysis via QC Method

**Figure 5:**
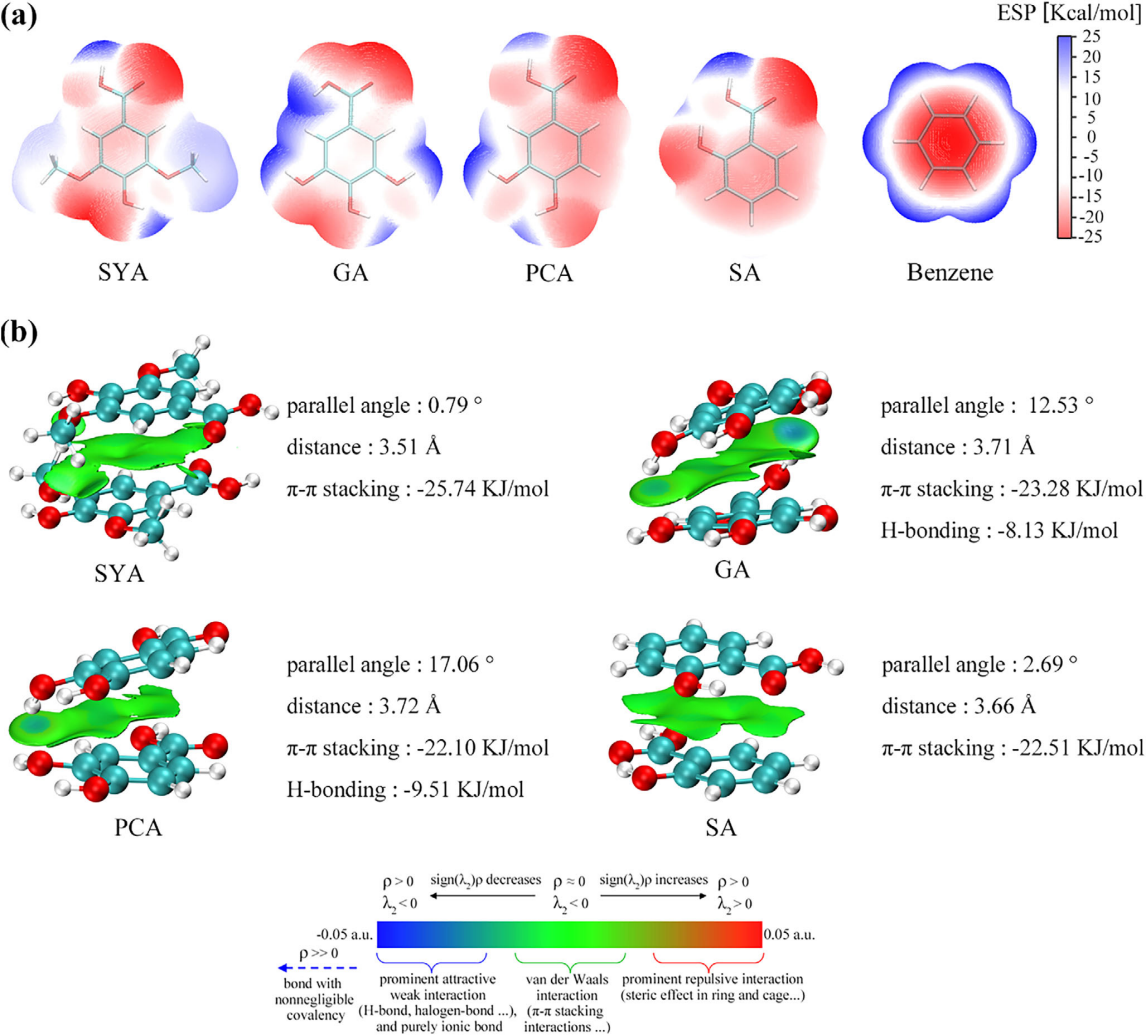
Analysis of π‐π stacking interaction. a) ESP on van der Waals surfaces of monophenols. b) Visualization of the structure and interactions of monophenol dimers (Color scale was adapted from literature^[^ ^102^ ^]^).

It is widely believed that the Electron‐withdrawing inductive effect of substituents enhances π‐π stacking interaction by lowering the electrostatic potential (ESP) of the aromatic rings’ π‐electron cloud.^[^ ^98^ ^]^ To explore this relationship between monophenol structures and π‐π stacking interactions, their ESPs were analyzed. An increase in the number of substituents lowered the aromatic rings’ ESP (SYA & GA < PCA < SA) (**Figure** **5**a), thereby strengthening π‐π stacking interactions (SYA & GA > PCA > SA). However, when the number of substituents was comparable, more the higher polar substituents did not enhance π‐π stacking interactions (SYA > GA) despite reducing the aromatic rings’ ESP (SYA > GA). This suggests that other factors might become more influential in governing π‐π stacking interactions when the number of substituents was similar.

To further investigate this observation, the structures and interaction forces of QC‐optimized monophenol dimers were analyzed. The higher polar substituents commonly found in monophenols (e.g., ‐COOH/‐OH) exhibited H‐bonding capacity (referring to those with dual functionality as H‐bonding donors and acceptors^[^ ^99^, ^100^, ^101^ ^]^), with more these substituents promoting the generation of H‐bonds weaker than π‐π stacking (Figure 5b). This, in turn, disrupted the parallel stacking of the aromatic rings, weakening the π‐π interactions (Based on the available data, H‐bonding energies exceeding 8.13 kJ mol⁻^1^ would disrupt the parallel stacking arrangement.). Although the intermolecular H‐bonds were relatively weaker and transient in a room‐temperature solution (Figure 4a), they were still significant enough to disrupt π‐π stacking interactions, especially when comparing monophenols with similar number of substituents where differences in ESP were minimal.

This analysis reveals a general pattern governing monophenols’ self‐assembly. Primarily driven by π‐π stacking interactions, the strength of self‐assembly was dictated by a balance between the number of substituents and the number of H‐bonding‐capable substituents on the aromatic ring. Increasing the total number of substituents strengthened π‐π stacking interactions. However, when the number of substituents was similar between monophenols, the presence of more H‐bonding‐capable substituents weakened π‐π stacking due to competing intermolecular H‐bonds. Thus, a predictive model for monophenol self‐assembly rates could be established by fitting a bivariate equation to the number of substituents and H‐bonding‐capable substituents and corresponding *S_calculated_* across characterized monophenols.

(2) $$S_{predicted}=21.10n_{sub}-6.34n_{H-bonds}-24.90\left(R^{2}=0.9909\right)$$

Here, *S_predicted_* represented the self‐assembly rate predicted from molecular structure. *n_sub_* denoted the number of substituents on monophenols, and *n_H‐bonds_* represented the number of H‐bonding‐capable substituents not involved in intramolecular H‐bonds (e.g., hydroxyl groups adjacent to carboxyl groups typically formed intramolecular H‐bonds). While this equation, derived from only four monophenol samples, requires further refinement with additional samples, it offers a valuable preliminary model for studying the interplay of molecular factors governing self‐assembly (*n_sub_*_&_ *n_H‐bonds_*). Furthermore, since the focus of this study was on monophenols in aqueous solutions, the model was only applicable to aqueous environments.

### 2.3 Separation of Monophenols in Binary‐Systems

While conventional NF separations often rely on differences in Stokes radius and charge, our systematic investigation into monophenols’ self‐assembly revealed a novel separation strategy. We proposed that by exploiting the distinct self‐assembly behaviors of different monophenols, effective separation can be achieved through NF based on size‐exclusion effects.

#### 2.3.1 Analysis of Monophenols’ Rejection

**Figure 6:**
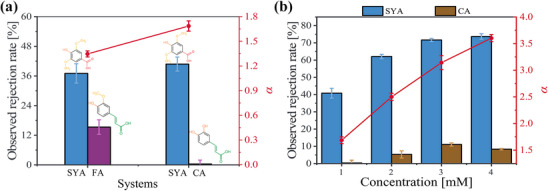
Analysis of separation for Monophenols in Binary‐systems. a) Observed rejection rates and separation factors of different monophenol binary systems. b) Observed rejection rates and separation factors of SYA−CA binary solutions at different concentrations.

To validate our proposed separation strategy, two binary mixtures of monophenols commonly coexisting in plants’ extracts (SYA − ferulic acid (FA) and SYA − CA)^[^ ^103^, ^104^, ^105^ ^]^ were subjected to NF. From structural perspective (Table 1), these monophenols have similar Stokes radius (FA > SYA > CA, Δ_max_ = 0.02), significantly reducing the impact of single‐molecule size on rejection behavior. Additionally, these three monophenols exhibited subtle structural differences. Both FA and CA had the same number of substituents, one fewer than SYA, but differed in their substituent groups, with FA being richer in methoxy groups and CA in hydroxyl groups. According to the Equation (2), their self‐assembly strengths were predicted to follow the order: SYA _(_ *S_predicted_*_= 46.82%)_ > FA _(_ *S_predicted_*_= 25.72%)_ > CA _(_ *S_predicted_*_= 19.38%)_. SYA consistently exhibited higher rejection rates in both binary systems, aligning with its stronger predicted self‐assembly (**Figure** **6**a). Furthermore, the SYA−FA mixture showed lower selectivity than the SYA−CA mixture, aligning with the smaller difference in self‐assembly strengths between SYA and FA. These results demonstrate that exploiting differences in self‐assembly behavior could enable effective separation of monophenols via NF. Moreover, concentration exerted non‐negligible impacts on monophenols’ NF separation induced by self‐assembly differences. Due to the concentration‐dependent enhancement of the self‐assembly effect, an increase in concentration intensified impact of self‐assembly on the NF separation of binary monophenol systems, leading to an increase in selectivity (Figure 6b). Intriguingly, Long‐term filtration tests reveal that the accumulation of monophenol with stronger self‐assembly in the retentate amplified both concentration polarization and self‐assembly effects. These two effects counteract each other in influencing rejection, resulting in marginal declines in the rejection of monophenol with stronger self‐assembly and stable selectivity (Figure S7, Supporting Information)​.

To validate the predictions of self‐assembly strength, MDS were used to study the self‐assembly behavior of the two monophenol mixtures.

#### 2.3.2 Analysis of Self‐Assembly Behavior

**Figure 7:**
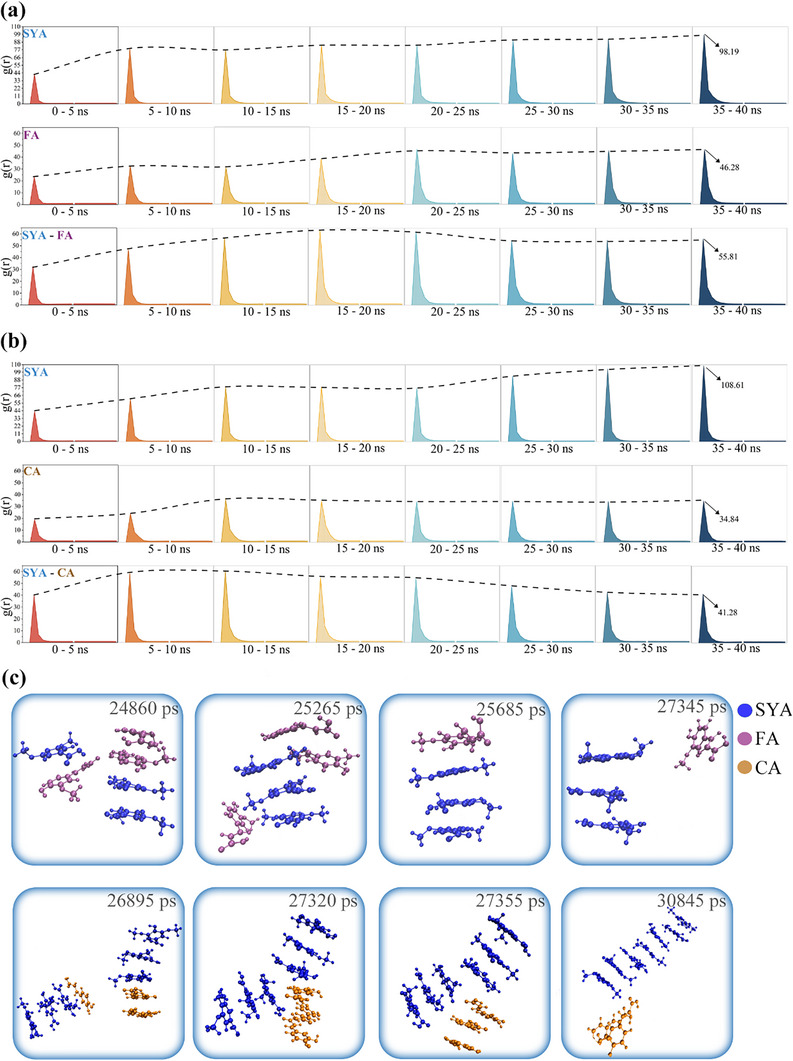
Analysis of self‐assembly behavior in binary‐systems. a) RDFs between monophenols in SYA–FA system every 5 ns during the production runs. b) RDFs between monophenols in SYA–CA system every 5 ns during the production runs. c) Typical snapshots of competitive self‐assembly processes among different monophenols during the production runs.

The RDFs between monophenols were analyzed every 5 ns during the production runs to dynamically characterize their self‐assembly behavior. **Figure** **7**a,b shows that the self‐assembly effect followed the order SYA > FA > CA, fully confirming our prediction. Notably, different monophenols could self‐assembly with each other. However, SYA's stronger self‐assembly dominated, replacing inter‐component self‐assemblies (Figure 7c) and causing their RDFs to peak and then gradually decrease. And compared to FA, CA's greater number of hydroxyl groups weakened its self‐assembly with SYA, making it more easily displaced. The π‐π stacking (Figure S8, Supporting Information) and H‐bonding (Figure S9, Supporting Information) analyses further reveal that SYA's stronger π‐π interactions were due to more substituents, while CA showed a greater tendency to form H‐bonds, weakening its self‐assembly compared to FA.

MDS of self‐assembly in SYA‐CA binary solutions at different concentrations revealed that increasing concentration significantly raised the coordination numbers (CNs) between monophenol molecules (Figure S10, Supporting Information). Furthermore, the disparity in CNs among different monophenol species became more pronounced. These results indicate that elevated concentration not only enhanced the self‐assembly propensity of monophenols, but also amplified the differences in self‐assembly between distinct species, with those exhibiting stronger self‐assembly showing more marked enhancement.

In summary, the reason the selectivity between SYA and FA was lower than between SYA and CA was due to CA's weaker self‐assembly, both among CA molecules and between CA and SYA. And elevated concentrations amplified the divergence in self‐assembly effect among monophenol Species. The Species with stronger self‐assembly effect exhibited more pronounced enhancement in self‐assembly effect, thereby improving separation selectivity.

### 2.4 Discussion on the impact of Self‐Assembly on Monophenols Separation by NF

**Figure 8:**
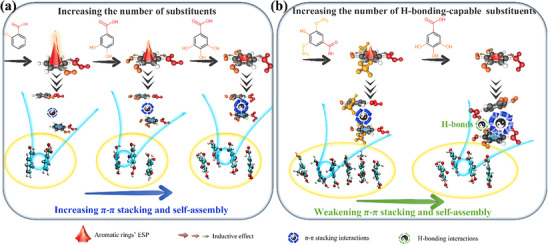
Mechanism diagram of the impact of substituents on monophenol self‐assembly. a) Impact of the number of substituents on monophenol self‐assembly. b) Impact of the number of H‐bonding‐capable substituents on monophenol self‐assembly.

This study highlights the significant impact of monophenols’ self‐assembly on NF separation. Self‐assembly is common among monophenols, with stronger self‐assembly leading to larger and more numerous self‐assemblies that are more easily rejected by membranes. Monophenols’ self‐assembly is primarily driven by π‐π stacking interaction, closely tied to molecular structure. Specifically, an increase in the number of substituents lowers the aromatic rings’ ESP, strengthening π‐π stacking interactions and promoting self‐assembly (**Figure** **8**a). When comparing monophenols with similar number of substituents, those with more H‐bonding‐capable substituents tend to form intermolecular H‐bonds, disrupting π‐π stacking and weakening self‐assembly (Figure 8b). This established structure−self‐assembly relationship not only elucidates the mechanisms and factors governing monophenols’ self‐assembly, but also provides a foundation for designing targeted separation strategies based on predictable size‐exclusion effects arising from differential self‐assembly behavior.

While this study investigates the self‐assembly behavior of monophenols, it is crucial to recognize the broader significance of this phenomenon in membrane processes. Given the prevalence of weak interactions in solution systems, self‐assembly may likely occur with other molecules as well. Therefore, when evaluating the influence of solute physicochemical properties (e.g., *pKa*, *logD*, Stokes radius) on rejection, it is crucial to consider potential self‐assembly effects. By elucidating the fundamental rules of their self‐assembly driving forces, we can derive patterns in self‐assembly and thereby predict the corresponding rejection behaviors.

Exploiting differences in size‐exclusion arising from molecular self‐assembly offers a promising new avenue for membrane separation of diverse molecules. However, it is important to acknowledge that self‐assembly is influenced not only by the intrinsic chemical structures of molecules, but also by environmental factors (e.g., pH, concentration, temperature, solvent types) and the membrane.^[^ ^83^, ^84^, ^85^, ^86^, ^106^, ^107^, ^108^, ^109^ ^]^ Therefore, realizing controlled and predictable membrane separations driven by differences in molecular self‐assembly requires further investigation into these contributing factors.

## 3 Conclusions

This study unveiled a novel paradigm for predicting and manipulating the rejection behavior of monophenols during NF, establishing a direct link between molecular self‐assembly and membrane separation performance. Through a synergistic combination of experimental and computational approaches, the self‐assembly of monophenols, primarily driven by π‐π stacking interactions, was demonstrated to significantly influence their rejection rates. Importantly, a clear structure−self‐assembly relationship was established, wherein the number and H‐bonding capacity of substituents on the aromatic ring were found to dictate the strength of self‐assembly. This insight enabled the development of a predictive model for monophenols’ self‐assembly, which was subsequently validated through membrane separation experiments using binary mixtures. This study significantly advanced membrane separation science by demonstrating that predictable solute self‐assembly can be harnessed for selective separation, paving the way for the rational design of novel separation strategies for a wide range of similarly sized molecules.

## 4 Experimental Section

### Chemicals and Reagents

Six monophenols were chosen as model solutes in the study due to their systematic variations in the number of substituents and H‐bonding‐capable substituents. SYA, GA, PCA, CA, and FA were sourced from Nantong Feiyu Biotechnology Co., Ltd., China, and SA was obtained from Shyuanye Biotechnology Co., Ltd., China. Table 1 summarizes their key physicochemical parameters. Ultrapure water for solution preparation was generated using a Milli‐Q system (Millipore, USA). High performance liquid chromatography (HPLC) grade methanol and acetonitrile were purchased from Hanbang Technology Co., Ltd.

### Membranes Characterization

Two commercial NF membranes (NF90 (Dow Filmtech, USA), NF270 (Dow Filmtech, USA)) were employed in this investigation. All membranes were acquired as flat sheets and cut into circular filters with a 7.3 cm diameter before use, providing an effective surface area of 41.8 cm^2^. The key properties of the membranes are listed in **Table** **2**.

**Table 2:** The key properties of NF membranes.

Membrane	NF90	NF270
Manufacturer ^a)^	Dow Filmtech	Dow Filmtech
Material ^a)^	Polyamide (PA)	Polypiperazine‐amide (pip‐PA)
Max pressure [bar] ^a)^	41	41
Max temp [°C] ^a)^	45	45
*pH* range ^a)^	2–11	3–10
Salt rejection [%] ^a)^	98.7% MgSO_4_	97% MgSO_4_
MWCO [Da] ^b)^	100–200	200–400
Pore radius [nm] ^c)^	0.36	0.44
Water permeability [L (m⁻^2^ h⁻^1^ bar)] ^d)^	8.68	13.04

^a)^ Information from the manufacturer, which the salt rejections were measured when 2 g L⁻^1^ MgSO4 was used as the feed liquid;

^b)^ Data cited from the literatures^[^ ^110^, ^111^, ^112^ ^]^;

^c)^ Data cited from the literatures^[^ ^113^, ^114^ ^]^;

^d)^ Before experiment, membranes were compacted with Milli‐Q water at 4 bar for at least 30 min. The pure water permeability was measured under the conditions of 25 °C and 2 bar.

### Membrane Filtration Experiments

A dead‐end filtration cell (UFSC40001, Millipore, USA) equipped with nitrogen pressure and a magnetic stirring paddle was employed for the experiments. New membranes were pre‐treated by soaking in Milli‐Q water for 24 h to remove preservatives. Before each experiment, membranes were compacted with Milli‐Q water at 4 bar for at least 30 min until the flux stabilized. During the experiment, temperature (25 °C), pressure (2 bar), and stir speed (300 rpm) were maintained constant.

### Membrane Filtration Experiments—NF of Monophenols in Unary‐Systems

Four 300 mL of monophenol solutions (SYA, GA, PCA, SA) at original pH and 10 mmol L⁻^1^ were filled to membrane modules (NF270, NF90). During the filtration processes, 200 µL of permeate was taken at 2, 4, 6, 8, 10, and 20 min. Membrane filtration processes continued until the permeate volume reached 150 mL (end station) and the final samples were collected. Each experiment was repeated at least three times.

The dynamic rejection rate (*R_d_*) was calculated using the following equation:

(3) $$R_{d}\left(\%\right)=\left(1-\frac{C_{i}}{C_{f}}\right)\cdot 100\%$$

Where *C_i_* represented the solute concentration in the permeate at 2, 4, 6, 8, 10, and 20 min, as well as when the total permeate volume reached 150 mL. *C_f_* represented the solute concentration in the feed solution.

The observed rejection rate (*R_obs_*) was calculated by^[^ ^115^ ^]^:

(4) $$R_{obs}\left(\%\right)=\left(1-\frac{C_{p}}{C_{f}}\right)\cdot 100\%$$

Where *C_p_* was the solute concentration in the permeate when the total permeate volume reached 150 mL, while *C_f_* was the solute concentration in the feed solution.

### Membrane Filtration Experiments—NF Separation of Monophenols in Binary Systems

Two 300 mL of binary monophenol mixtures (SYA – FA, SYA − CA), at original pH and 1 mmol L⁻^1^ for each monophenol, were introduced into the membrane modules (NF270). The filtration processes continued until the permeate volume reached 150 mL, and both permeate and feed solutions were collected. Each experiment was repeated at least three times.

The selectivity (*α*) was considered as follows:

(5) $$Selectivity\alpha =\frac{X_{p}/Y_{p}}{X_{f}/Y_{f}}$$

Where *X_p_* and *Y_p_* were the concentrations of two types of solutes in the permeate, respectively; *X_f_* and *Y_f_* were the concentrations of two types of solutes in the feed, respectively.

### Membrane Filtration Experiments—NF Separation of Binary Monophenol Mixtures with Different Concentrations

300 mL of binary monophenol mixtures (SYA−CA) at concentrations of 1, 2, 3, and 4 mmol L^−1^ for each monophenol were introduced into the membrane modules (NF270). The filtration processes continued until the permeate volume reached 150 mL, and both permeate and feed solutions were collected. Each experiment was repeated at least three times.

### Membrane Filtration Experiments—Long‐Term Separation Performance Testing of NF for Binary Monophenol Mixtures

400 mL of binary monophenol mixtures (SYA − CA), at original pH and 1 mmol L⁻^1^ for each monophenol, were filled into membrane modules (NF270). During the filtration processes, 200 µL of permeate was taken at 30‐min intervals. Membrane filtration processes continued until the permeate volume reached 300 mL (end station), and the final samples were collected. Each experiment was repeated at least three times.

### Static Adsorption Experiment

The new membranes (NF270, NF90) were soaked in Milli‐Q water for 24 h to remove preservatives. It was then fabricated into an attached film chip^[^ ^116^ ^]^ and installed in the quartz crystal microbalance with dissipation (QCM‐D) sample chambers. Prior to the experiment, air was pumped into the sample chambers at 150 µL min⁻^1^ until the baseline stabilized, followed by deionized water at the same rate. During the experiment, four 10 mmol L⁻^1^ monophenol solutions (SYA, GA, PCA, SA) were each pumped into the chamber at 100 µL min⁻^1^ until the baseline stabilized, after which data collection was stopped.

### Experimental Characterization of Monophenol Self‐Assemblies

Dynamic light scattering (DLS, Malvern‐Zetasizer Nano ZS 90, Malvern Panalytical, England) was utilized to determine hydrodynamic diameters of self‐assemblies in 10 mmol L⁻^1^ monophenol solutions (SYA, GA, PCA, SA) at 25 °C. TEM (HT‐7800, Hitachi High‐Tech, Japan) was employed to characterize the morphology of the self‐assemblies in 10 mmol L⁻^1^ monophenol solutions​. UV–vis spectrophotometry (UV‐2401 PC, Shimadzu, Japan) was performed to analyze the absorption spectra of monophenol solutions (SYA, GA, PCA, SA) at 0.1 and 10 mmol L⁻^1^ concentrations. A SEM (QUANTA 250 FEG, Thermo Fisher, USA) was used to examine the membrane surfaces morphology after NF.

### Analytical Methods—HPLC

Monophenol concentrations were determined using a HPLC system (Agilent Technologies, 1260 Series, USA) equipped with a UV detector. Chromatographic separations were performed on ZORBAX SB‐C18 (4.6 × 250 mm, 5 µm, Agilent Technologies Co., Ltd., America) and Hypersil QDS2 columns (4.6 × 250 mm, 5 µm, Dalian Elite Analytical Instruments Co., Ltd., China) with different elution methods tailored to each monophenol (Experimental Section 1, Supporting Information). All samples were filtered through 0.22 µm cellulose acetate filters before injection and diluted 1:5 with Milli‐Q water. The concentrations were quantified by analytical interpolation using a standard calibration curve based on the peak area.

### Analytical Methods—QCM‐D

The QCM‐D used in the experiment was obtained from Biolin Scientific (Sweden), and the sensor used was gold‐coated quartz sensors (QSX 301). The software QSoft401 was used to record the changes in the resonance frequency and energy dissipation. The QSense‐Dfind software was used to calculate the adsorption mass.

### Modeling

**Figure 9:**
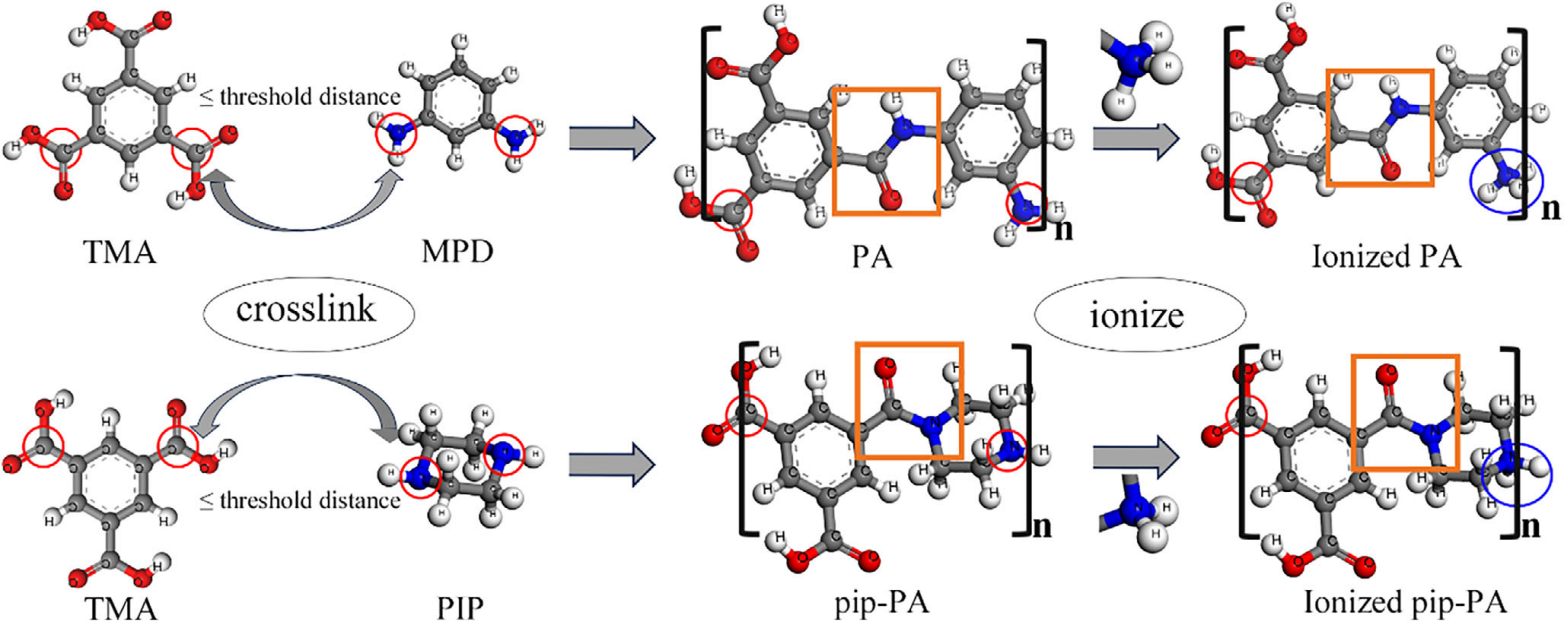
Diagram of the construction process for PA and pip‐PA membranes, where gray represents C, blue represents N, white represents H, and red represents O.

The NF270 and NF90 membranes were constructed using heuristic crosslink. Table 2 shows that the materials are PA for the NF90 and pip‐PA for the NF270. The PA membrane was constructed through the heuristic crosslink of *m‐*phenylenediamine (MPD) and trimesic acid (TMA), while the pip‐PA membrane was constructed through the heuristic crosslink of piperazine (PIP) and TMA. As shown in **Figure** **9**, when the distance between reacting atoms fell within the reactive threshold, hydroxyl and H atoms were removed, and a new bond was formed between them. The reactive threshold would be gradually increased to 13 nm as cross‐linking progressed. Given the SA solution's original pH of 2.6, the unreacted N atoms in the models must be ionized to carry a positive charge before introducing the SA solution (Figure S1, Supporting Information).^[^ ^117^, ^118^ ^]^ The physical parameters of the models and their corresponding reference values were summarized in Table S1 (Supporting Information).

Each monophenol existed in two forms at their respective original pH: the neutral and ionized (Figure S11, Supporting Information). The neutral was optimized using density functional theory (DFT) with the B3LYP‐D3(BJ)/def2TZVP level, while the ionized employed B3LYP‐D3(BJ)/maTZVP. All geometry optimizations were conducted in implicit water and followed by vibrational analyses using consistent computational settings (theory/basis set/solvent) to confirm no imaginary frequencies. Atomic charge was fitted using Multiwfn^[^ ^119^ ^]^ to obtain advanced restrained electrostatic potential (RESP2) charge and the solvent‐environment parametrization of RESP2 charges was performed using wavefunction files obtained from single‐point energy calculations on monophenols in implicit water, and the topology files were generated with Sobtop. Water was represented using the three‐point potential model. Details of ion models force field parameters are provided in Table S2 (Supporting Information).

### MDS Details—Simulation Setting

MDS were performed and analyzed using GROMACS 2021.7, and visualizing the trajectories was conducted with VMD. The molecular topology was defined based on the general AMBER force field (GAFF). The cutoff distance for the non‐bonded Lennard‐Jones interaction was set at 1 nm. The Particle‐Mesh Ewald method was employed to calculate the long‐range electrostatic forces. The linear constraint solver algorithm was utilized to maintain covalent bonds involving H atoms at their equilibrium values. The NPT ensemble was employed in both the equilibrium and production runs in this study. The V‐rescale method was employed to control the temperature at 298.15 K. Additionally, the Berendsen and Parrinello‐Rahman methods were utilized to keep the NPT ensemble at 1 bar during the equilibrium and production runs, respectively.

### MDS Details—The Simulation of Absorption for Monophenols

In this study, the interaction energies between monophenols (SYA, GA, PCA, SA) and the membranes (PA, pip‐PA) were examined at their original pH. Given the effect of pH on solute ionization, the Henderson‐Hasselbalch equation^[^ ^120^ ^]^ was used to determine the neutral‐to‐ionized ratio for each monophenol. In each system, 20 monophenol molecules were positioned 1–2 nm from the membrane surfaces. The specific compositions of the systems are detailed in Table S3 (Supporting Information). All systems underwent 1 ns equilibrium runs to achieve stability (i.e., the overall energy and temperature exhibited small fluctuations) followed by 30 ns production runs for data collection. During equilibrium runs, 1 000 N forces were applied along all x, y, z axes to atoms in the membrane and solute to prevent structural deformation and relax the system to the desired thermodynamic state. The production runs comprised two sequential stages: the initial 20 ns run to bring the different monophenols to adsorption equilibrium on the membrane surface, followed by 10 ns runs to calculate the interaction between the adsorbed monophenols and the membrane.

### MDS Details—The Simulation of Self‐Assembly Behavior of Monophenols—Unary‐Systems

This study focused on the self‐assembly behavior of monophenols (SYA, GA, PCA, SA) in unary‐component solutions at their original pH. To simulate corresponding the original pH, the Henderson‐Hasselbalch equation was used to calculate the neutral‐to‐ionized ratio for each monophenol, with the corresponding hydrated protons added to the systems. The specific compositions of the systems are outlined in Table S4 (Supporting Information). Each system underwent a 1  ns equilibrium run followed by a 40 ns production run. Binary‐Systems: The primary focus of this simulation was to investigate the self‐assembly behavior of monophenols in mixed binary‐component solutions at the original pH. The specific compositions of the systems are detailed in Table S5 (Supporting Information). Other simulation settings were consistent with those in the subhead “Unary‐Systems.” Binary Systems with Different Concentrations: This simulation primarily investigates the impact of concentration on the self‐assembly behavior for monophenols in mixed binary‐component solutions at the original pH. Monophenols were introduced into different simulated systems in progressively increasing amounts. The specific compositions of the systems are detailed in Table S6 (Supporting Information). Other simulation settings were consistent with those in the subhead “Unary‐Systems.”

### QC Details—Visualization of the Electrostatic Potential of Monophenols

The structures of SYA, GA, PCA, and SA were optimized using DFT at the B3LYP‐D3(BJ)/def2TZVP level, generating wavefunction files. All geometry optimizations were conducted in implicit water and followed by vibrational analyses using consistent computational settings (theory/basis set/solvent) to confirm no imaginary frequencies. Multiwfn was used to fit ESP on van der Waals surface of the molecules based on the wavefunction files generated from geometry optimizations, and VMD was employed for visualization and plotting based on the fitted data.

### QC Details—Analysis of Monophenol Dimers

The monophenol dimers generated by MDS were selected as the initial structures and further optimized using DFT at the B3LYP‐D3(BJ)/def2TZVP level. All geometry optimizations were conducted in implicit water and followed by vibrational analyses using consistent computational settings (theory / basis set / solvent) to confirm no imaginary frequencies. Multiwfn was then employed for visualizing weak interactions between the monophenol dimers based on the independent gradient model and Hirshfeld partition theory.^[^ ^102^ ^]^ Additionally, the dispersion interactions between aromatic rings (the essence of π‐π stacking interaction) were quantitatively analyzed using the GAFF.^[^ ^121^ ^]^

## Conflict of Interest

The authors declare no conflict of interest.

## Supporting information



Supporting Information

## Data Availability

The data that support the findings of this study are available from the corresponding author upon reasonable request.
