# Supplementary material for: Predictable Self‐Assembly as an Unexplored Key Factor Influencing Membrane Separation: Insights from Monophenols
Source: Adv Sci (Weinh). 2025 Jun 20;12(34):e04322. doi: 10.1002/advs.202504322 (PMC12442694; doi:10.1002/advs.202504322)
Supplement: Supplementary file 1 — Supporting Information [file ADVS-12-e04322-s001.docx]

**SUPPORTING INFORMATION**

**Predictable Self-assembly as an Unexplored Key Factor Influencing Membrane Separation: Insights from Monophenols**

Qiuyu Han ^a, b^, Lu Yin ^a, b^, Tingting Mi ^a, b^, Qi Chen ^a, b^, Wanlin Ouyang ^a, b^, Liping Fan ^a, b^, Qinshi Wang ^a, b^, Yue Zhang ^a, b^, Zhishu Tang ^d^, Huaxu Zhu ^a, b^, Bo Li ^a, b, c,^ *

^a^ Jiangsu Collaborative Innovation Center of Chinese Medicinal Resources Industrialization, Nanjing University of Chinese Medicine, Nanjing 210023, China

^b^ Jiangsu Research Center of Botanical Medicine Refinement Engineering, Nanjing University of Chinese Medicine, Nanjing 210023, China

^c^ The First Clinical Medical College, Nanjing University of Chinese Medicine, Nanjing, 210023, China

^d^ School of Chinese Materia Medica, Beijing University of Chinese Medicine, Beijing 100029, China

* Corresponding author. Tel: 025-85811962. E-mail: Boli@njucm.edu.cn (B. Li)

Submitted to

*Advanced Science*

18 May 2025

1. **Supplementary Experimental Methods 1**

Elution methods for unary -systems (SYA, GA, PCA) and binary-systems (SYA-FA, SYA-CA): A ZORBAX SB-C18 was employed under the following operating conditions: mobile phase flow rate of 1 mL∙min^-1^, column temperature of 30 ℃, and pressure of 110 bar. The mobile phase was a mixture of methanol (solvent A) and 100:0.1 water/formic acid (v/v) (solvent B). A 21 min linear gradient elution was applied: 0 − 1 min 5 % A, 1 − 11 min 5 % − 15 % A, 11 − 21 min 15 % − 25 % A.

Elution methods for unary -system (SA): A Hypersil ODS2 was employed under the following operating conditions: mobile phase flow rate of 1 mL∙min^-1^, column temperature of 30 ℃, and pressure of 110 bar. The mobile phase was a mixture of acetonitrile (solvent A) and 100:0.1 water/ phosphoric acid (v/v) (solvent B). A 10 min isocratic elution was applied: 0 − 10 min 55 % A.

1. **Supplementary calculations equation 1**

The hydrophobic area rate was used to characterize the hydrophobicity of the solute, calculated as follows:

$$\begin{aligned} Hydrophobic area rate\left( \% \right)=\frac{Hydrophobic area}{solvent-accessible surface area}*100\#\left( S1 \right) \end{aligned}$$

**Table S1 The physical parameters of the NF membrane models and the reference values.**

| Physicochemical properties | PA membrane | Ref. | pip-PA membrane | Ref. |
| --- | --- | --- | --- | --- |
| Density [g/cm^3^] | 1.26 | 1.26 ^[1]^ | 1.27 | 1.24 ^[2]^ |
| O/N | 1.39 | 1.12 – 1.87 ^[3]^ | 1.29 | 1.19 – 1.42 ^[4-6]^ |
| Degree of crosslinking [%] | 83.07 | 70 – 90 ^[1,7-9]^ | 81.44 | 80 ^[10]^ |
| Thickness [nm] | 4.8 | ≥ 3 ^[11-13]^ | 4.8 | 5 - 7 ^[14]^ |

**Table S2 The information about ion models**

| **moleculetype** | | | | | | | | | | | | | | |
| --- | --- | --- | --- | --- | --- | --- | --- | --- | --- | --- | --- | --- | --- | --- |
| molname | | | | | | | | nrexcl | | | | | | |
| NA | | | | | | | | 1 | | | | | | |
| atomtypes | | | | | | | | | | | | | | |
| name | | at.num | | | mass | | | ptype | | sigma | | | epsilon | |
| Na | | 11 | | | 22.99 | | | A | | 3.32840e-1 | | | 1.15897e-1 | |
| atoms | | | | | | | | | | | | | | |
| id | at type | | | res nr | res name | | at name | | cg nr | | charge | | | mass |
| 1 | Na | | | 1 | NA | | NA | | 1 | | 1.00000 | | | 22.99 |
| **moleculetype** | | | | | | | | | | | | | | |
| molname | | | | | | | | nrexcl | | | | | | |
| CL | | | | | | | | 1 | | | | | | |
| atomtypes | | | | | | | | | | | | | | |
| name | | at.num | | | mass | | | ptype | | sigma | | | epsilon | |
| Cl | | 17 | | | 35.45 | | | A | | 4.40104e-1 | | | 4.18400e-1 | |
| atoms | | | | | | | | | | | | | | |
| id | at type | | | res nr | res name | | at name | | cg nr | | charge | | | mass |
| 1 | Cl | | | 1 | CL | | CL | | 1 | | -1.00000 | | | 35.45 |
| **moleculetype** | | | | | | | | | | | | | | |
| molname | | | | | | | | nrexcl | | | | | | |
| H3O+ | | | | | | | | 3 | | | | | | |
| atomtypes | | | | | | | | | | | | | | |
| name | | at.num | | | mass | | | ptype | | sigma | | | epsilon | |
| oh_H3O | | 8 | | | 15.999405 | | | A | | 3.066473e-1 | | | 8.803136e-1 | |
| ho_ H3O | | 1 | | | 1.007941 | | | A | | 0 | | | 0 | |
| atoms | | | | | | | | | | | | | | |
| id | at type | | | res nr | res name | | at name | | cg nr | | charge | | | mass |
| 1 | ho_ H3O | | | 1 | H3O | | H1 | | 1 | | 0.499806 | | | 1.007941 |
| 2 | oh_H3O | | | 1 | H3O | | O2 | | 2 | | 0.499419 | | | 15.999405 |
| 3 | ho_ H3O | | | 1 | H3O | | H3 | | 3 | | 0.499806 | | | 1.007941 |
| 4 | ho_ H3O | | | 1 | H3O | | H4 | | 4 | | 0.499806 | | | 1.007941 |
| bonds | | | | | | | | | | | | | | |
| atom_i | | | atom_j | | | functype | | | r0 (nm) | | | k (KJ/mol/nm^4^) | | |
| 1 | | | 2 | | | 2 | | | 0.098047 | | | 2.0656e+7 | | |
| 2 | | | 3 | | | 2 | | | 0.098047 | | | 2.0656e+7 | | |
| 2 | | | 4 | | | 2 | | | 0.098047 | | | 2.0656e+7 | | |
| angles | | | | | | | | | | | | | | |
| atom_i | | atom_j | | | atom_k | | | functype | | a0 (Deg.) | | | k (KJ/mol) | |
| 1 | | 2 | | | 3 | | | 2 | | 110.35605 | | | 450.00 | |
| 1 | | 2 | | | 4 | | | 2 | | 110.35605 | | | 450.00 | |
| 2 | | 3 | | | 4 | | | 2 | | 110.35605 | | | 450.00 | |

**Table S3 Arrangement of simulation of absorption for monophenols**

| Systems | Number of ionized species | Number of neutral species | Number of sodium ions | Number of  water molecules |
| --- | --- | --- | --- | --- |
| SYA_(PA)_ | 16 | 4 | 4 | 19324 ± 10 |
| GA_(PA)_ | 17 | 3 | 3 | 19376 ± 10 |
| PCA_(PA)_ | 18 | 2 | 2 | 19381 ± 5 |
| SA_(PA)_ | 12 | 8 | 3 | 19665 ± 5 |
| SYA_(pip-PA)_ | 16 | 4 | 4 | 20943 ± 2 |
| GA_(pip-PA)_ | 17 | 3 | 3 | 20988 ± 4 |
| PCA_(pip-PA)_ | 18 | 2 | 2 | 21000 ± 3 |
| SA_(pip-PA)_ | 12 | 8 | 0 | 21417 ± 12 |

**Table S4 Arrangement of simulation of self-assembly behavior of Monophenols in unary -systems**

| Systems | SYA solution | GA solution | PCA solution | SA solution |
| --- | --- | --- | --- | --- |
| pH | 3.3 | 3.2 | 3.2 | 2.6 |
| Number of neutral species | 122 | 127 | 135 | 91 |
| Number of ionized species | 28 | 23 | 15 | 59 |
| Number of sodium ions | 28 | 23 | 15 | 59 |
| Number of hydrated protons | 3 | 3 | 3 | 13 |
| Number of chloride ions | 3 | 3 | 3 | 13 |
| Number of water molecules | 271481 | 271803 | 271856 | 271851 |

**Table S5 Arrangement of simulation of self-assembly behavior of Monophenols in Binary -systems.**

| Systems | SYA-FA mixed solution | SYA-CA mixed solution |
| --- | --- | --- |
| pH | 3.6 | 3.6 |
| Number of ionized SYA | 32 | 32 |
| Number of ionized FA | 30 | 0 |
| Number of ionized CA | 0 | 36 |
| Number of neutral SYA | 68 | 68 |
| Number of neutral FA | 70 | 0 |
| Number of neutral CA | 0 | 64 |
| Number of sodium ions | 62 | 68 |
| Number of hydrated protons | 1 | 1 |
| Number of chloride ions | 1 | 1 |
| Number of water molecules | 270981 | 271073 |

**Table S6 Arrangement of simulation of self-assembly behavior of Monophenols in Binary systems at different concentrations.**

| Systems | Low concentration | Medium concentration | High concentration |
| --- | --- | --- | --- |
| pH | 3.6 | 3.6 | 3.6 |
| Number of neutral SYA | 34 | 68 | 136 |
| Number of ionized SYA | 16 | 32 | 64 |
| Number of neutral CA | 32 | 64 | 128 |
| Number of ionized CA | 18 | 36 | 72 |
| Number of sodium ions | 34 | 68 | 136 |
| Number of hydrated protons | 1 | 1 | 1 |
| Number of chloride ions | 1 | 1 | 1 |
| Number of water molecules | 272233 | 271073 | 268398 |


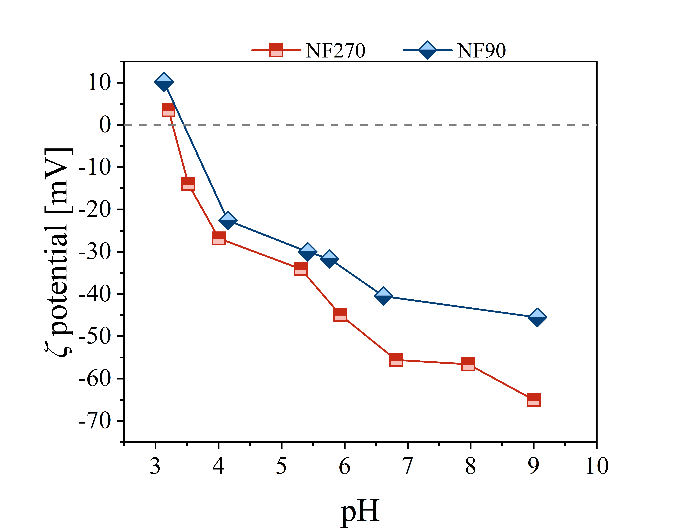


**Figure S1. The ζ potential of NF270 and NF90 as a function of pH. (A solution of 0.001 mol/L KCl was utilized as the background solution)**


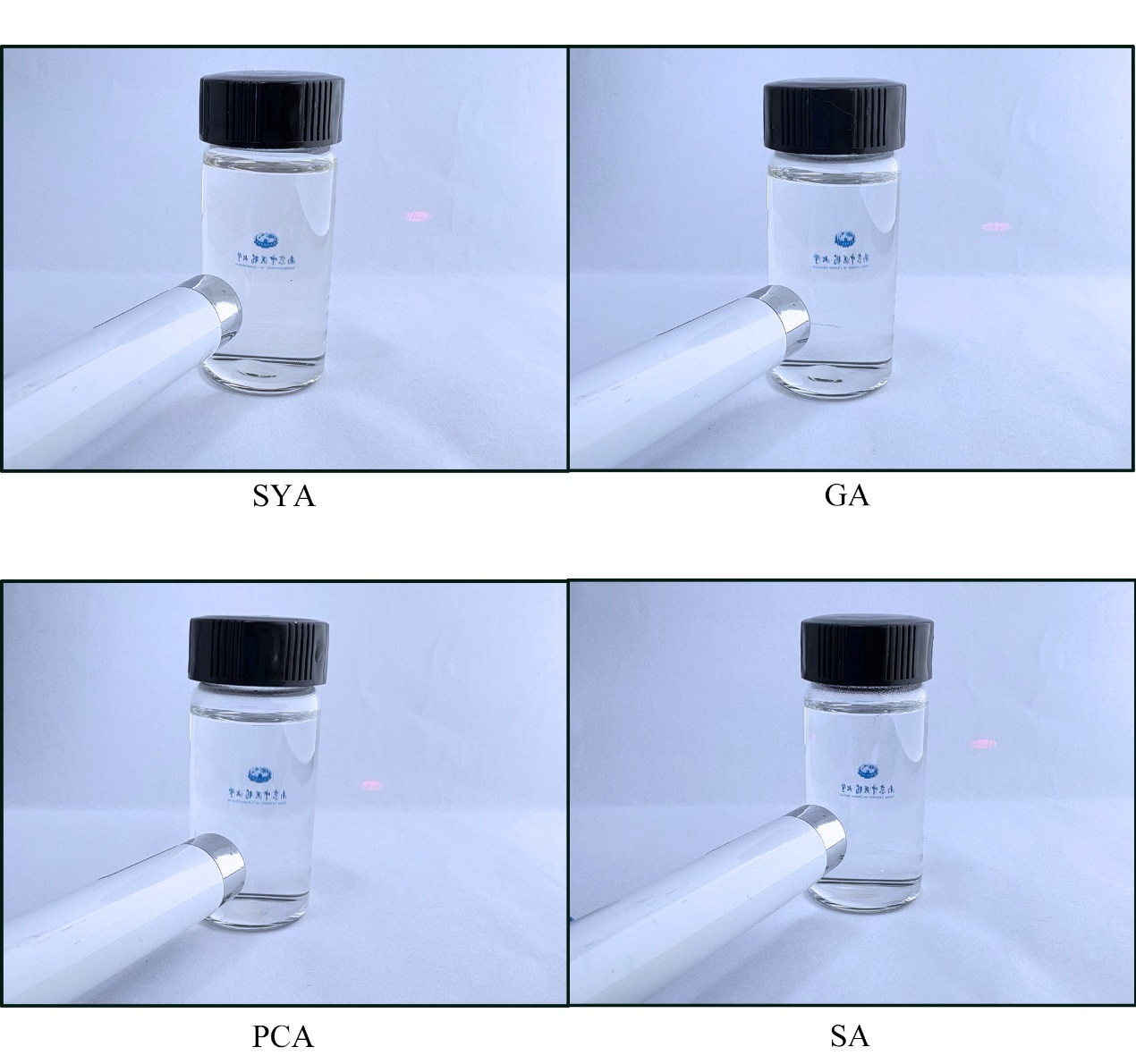


**Figure S2. Tyndall effect test for four feed solutions.**


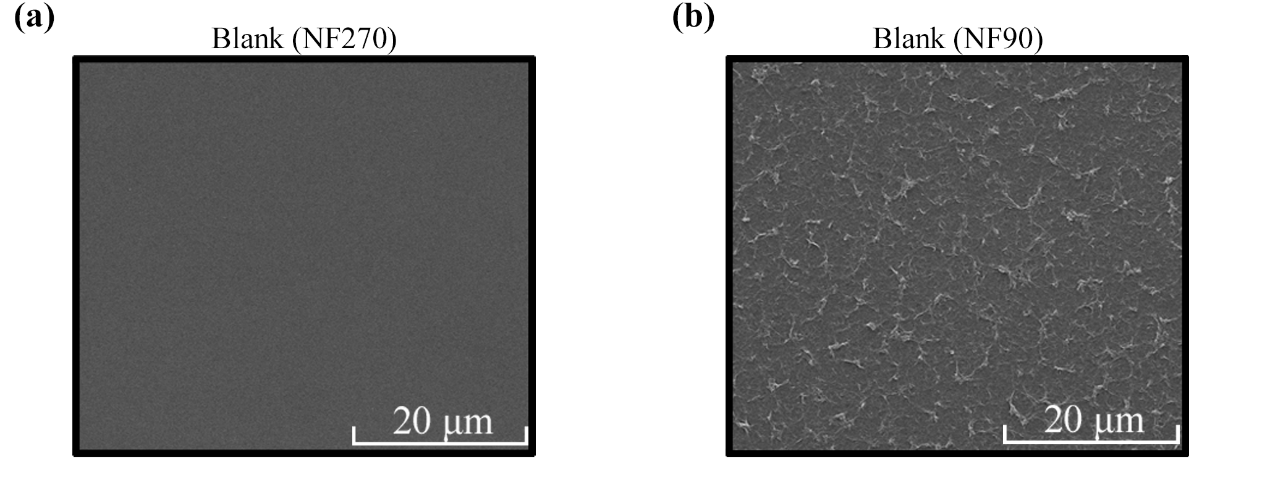


**Figure S3. SEM image of the blank control membranes surfaces (a) NF270. (b) NF90.**


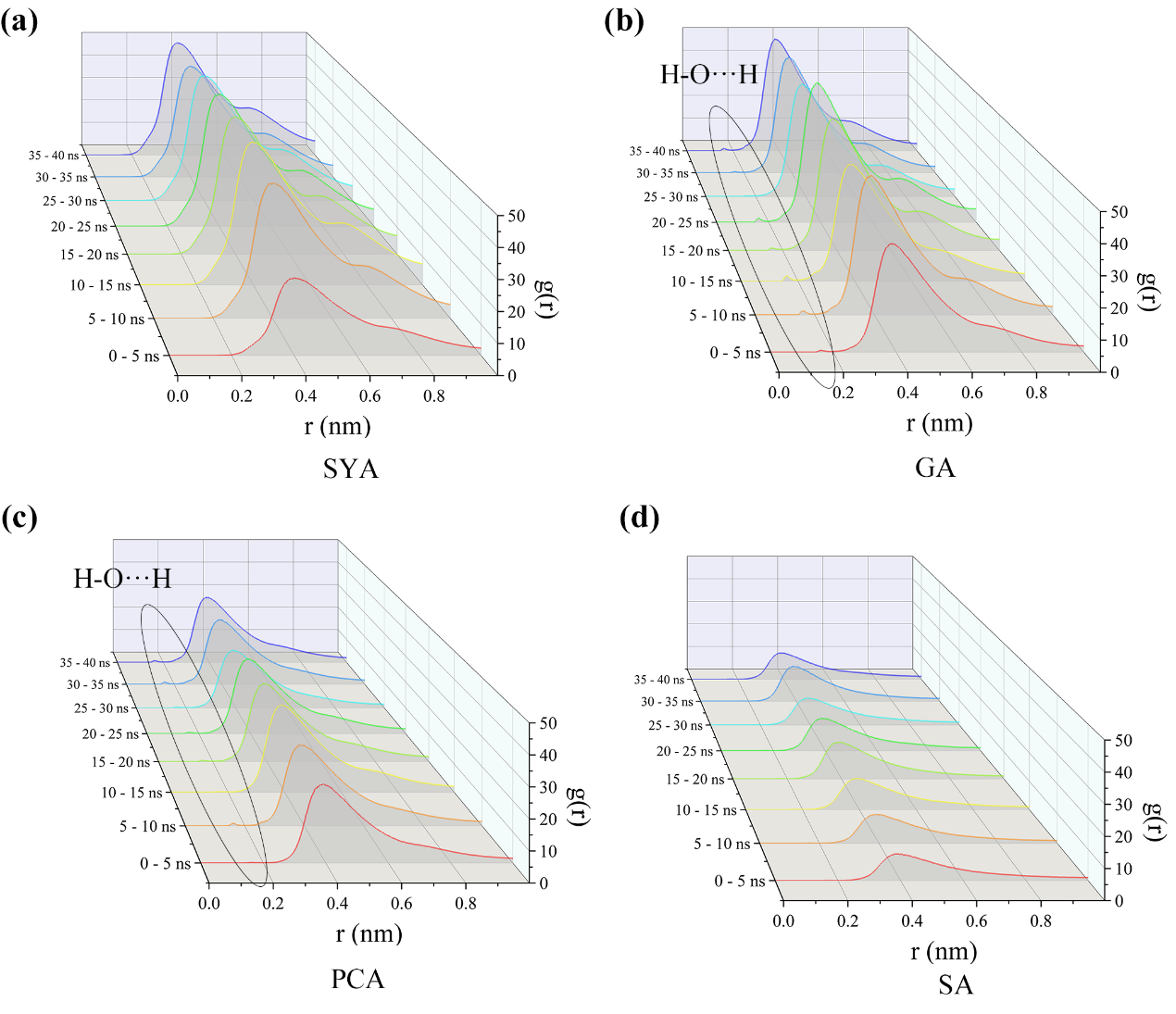


**Figure S4. The O − H RDFs of monophenols every 5 ns during the production runs (a) SYA. (b) GA. (c) PCA. (d) SA. (excl = 3000, bin = 0.002, *r_max_* = 1)**


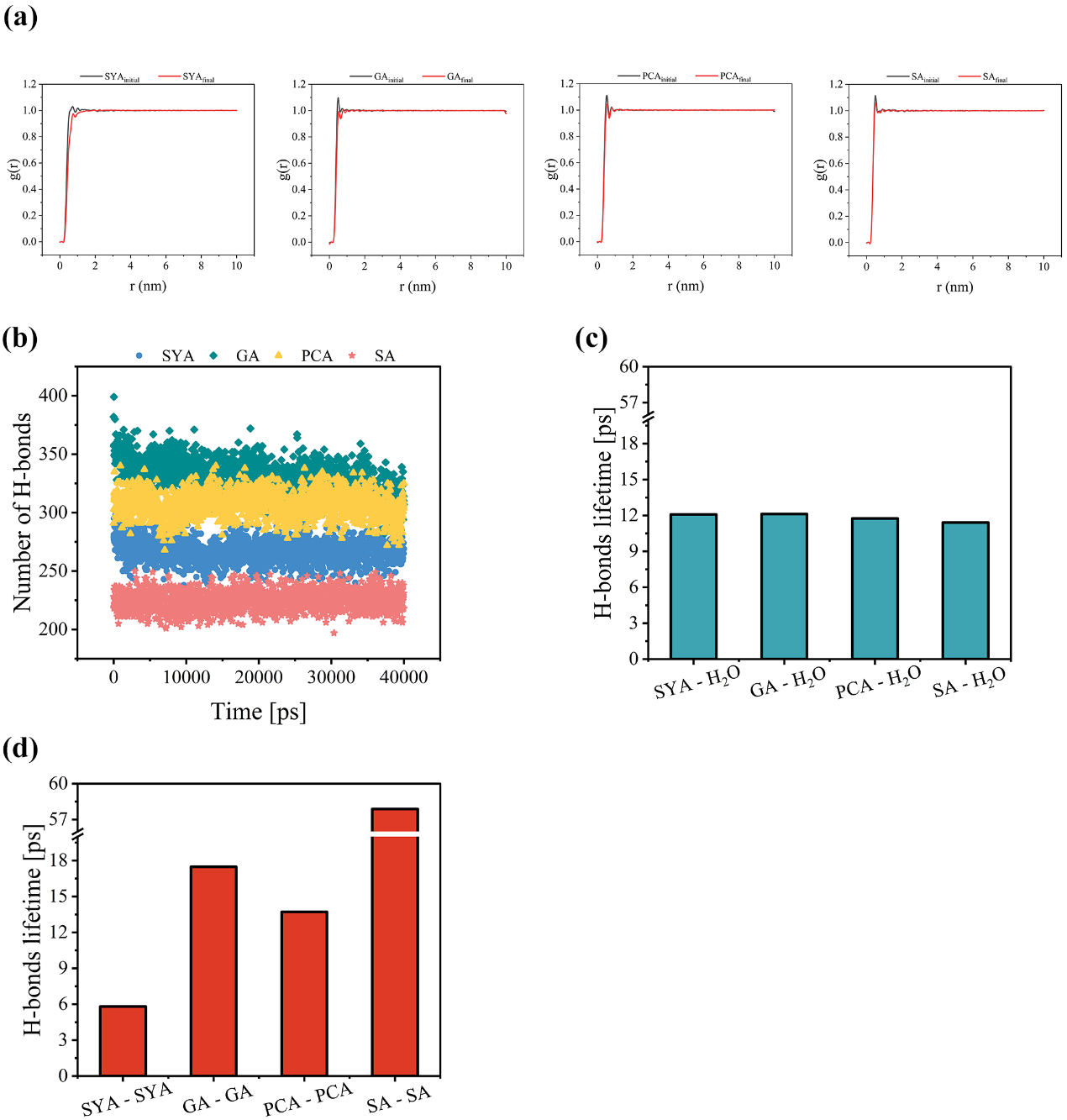


**Figure S5.** **Analysis of hydrogen bonding interactions between water and monophenols. (a) RDFs of monophenol − water during the initial 100 ps and final 100 ps of the production runs. (b) Variation in the number of H-bonds between monophenols and water over the 40 ns production runs. (c) H-bonding lifetime between monophenols and water. (d) intermolecular and intramolecular H-bonding lifetime in monophenols**


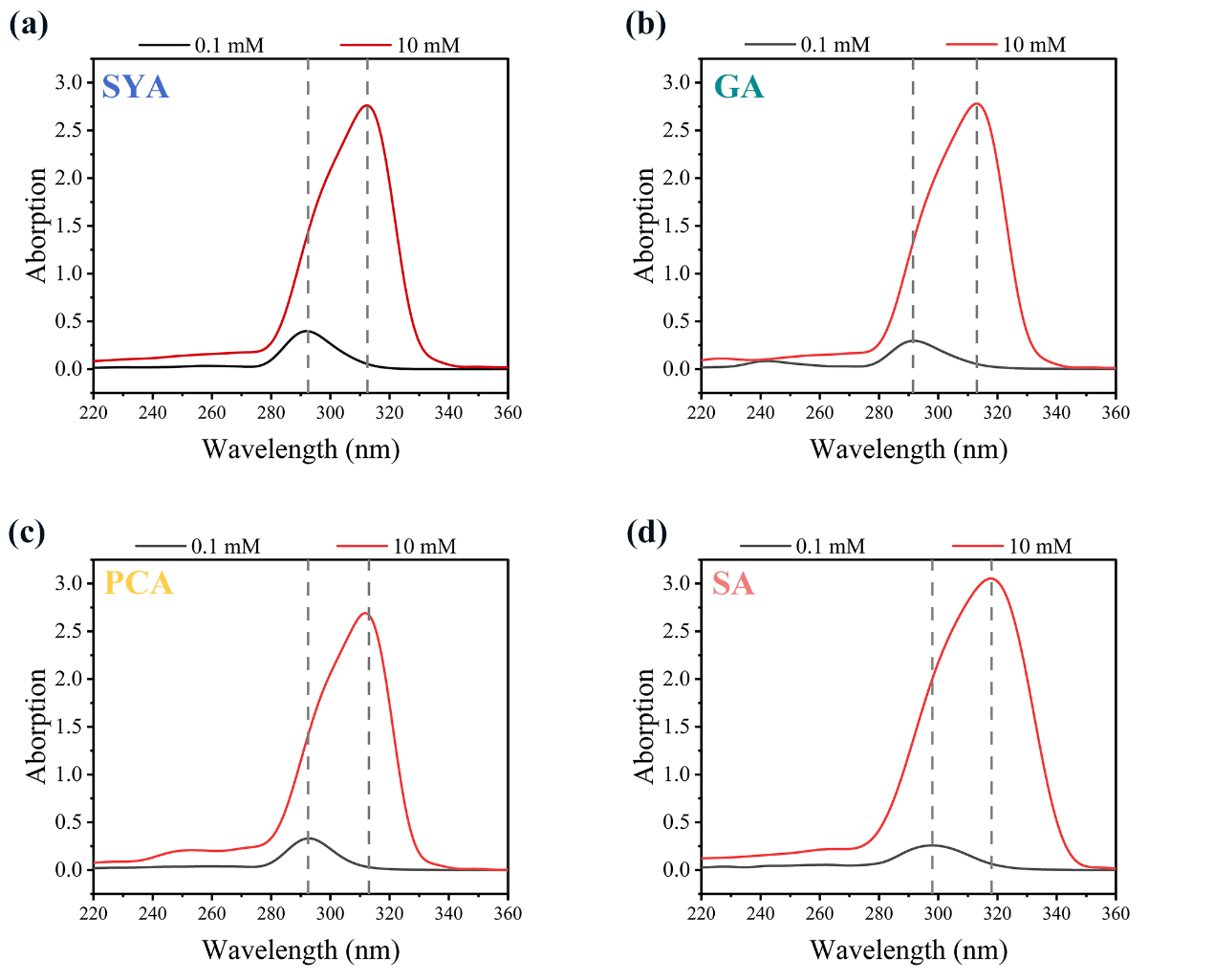


**Figure S6. UV-vis spectra of monophenol solutions at varying concentrations (a) SYA. (b) GA. (c) PCA. (d) SA.**


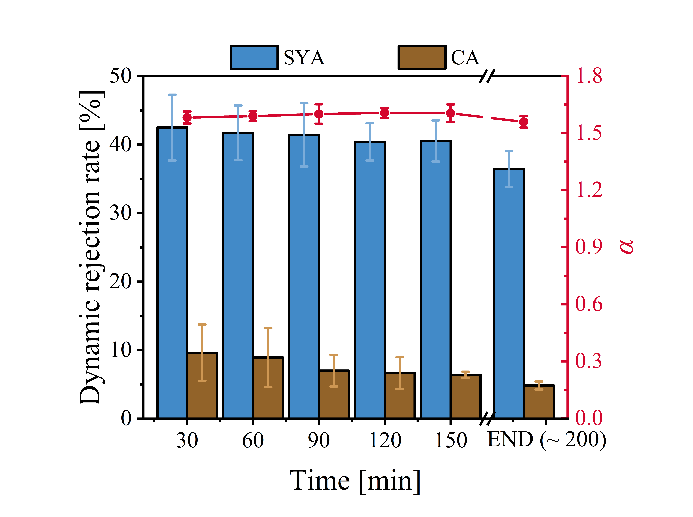


**Figure S7. Dynamic rejection rates and separation factors of the SYA-CA binary system during long-term NF.**


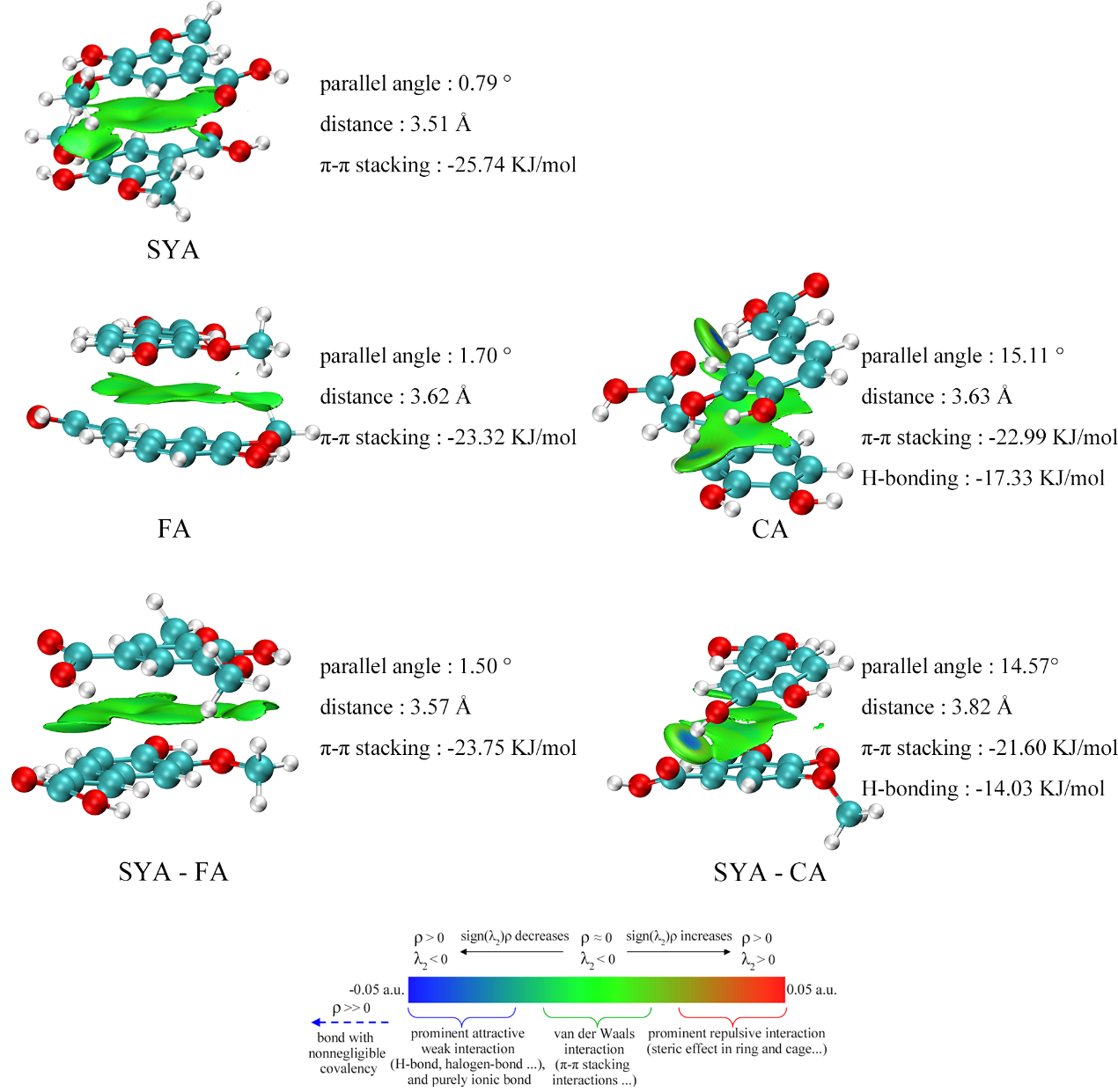


**Figure S8.** **Visualization of the structure and interactions of** **monophenol dimers.**


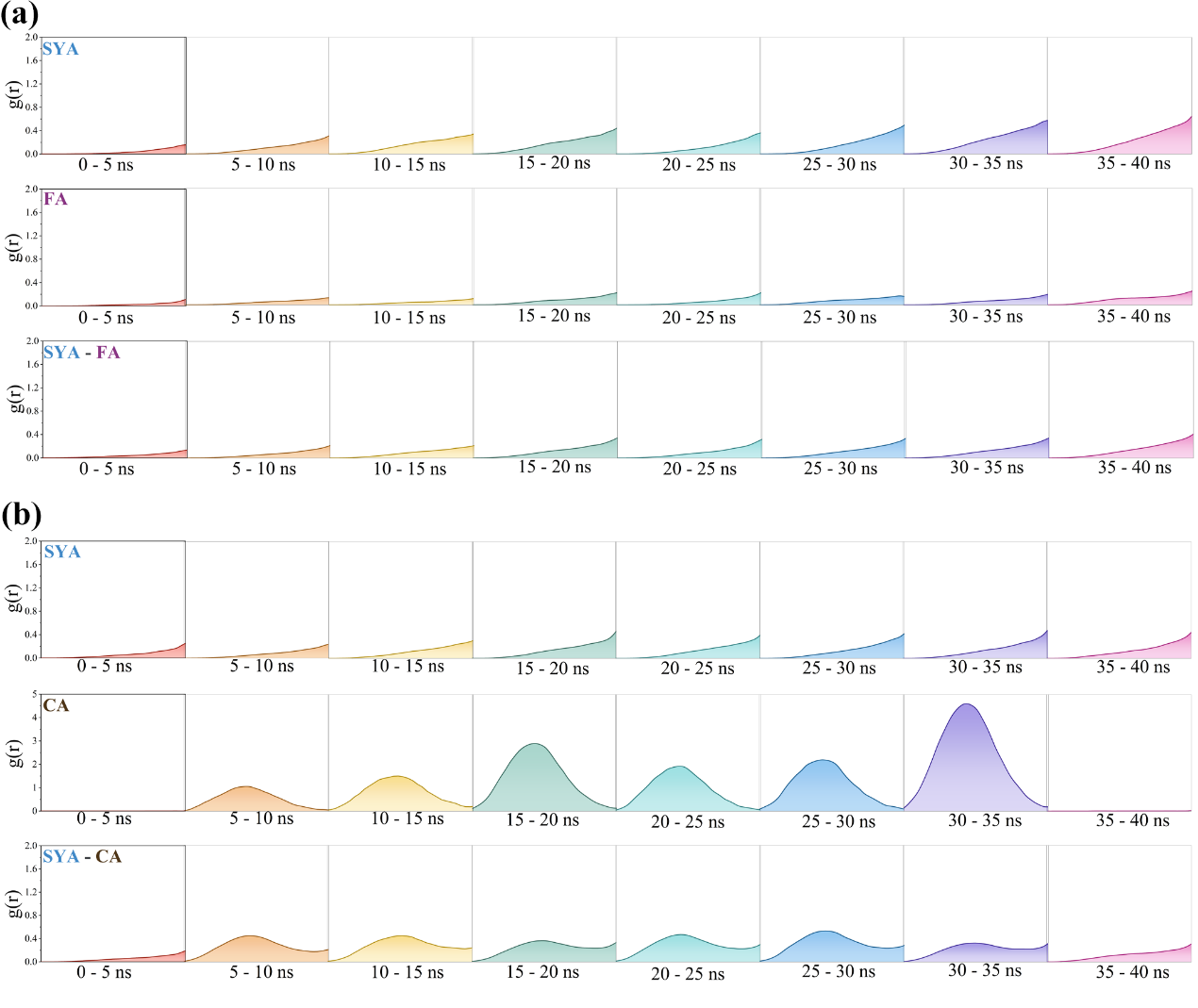


**Figure. S9. The RDFs of intermolecular H-bonds in different systems every 5 ns during the production runs (a) SYA – FA system. (b) SYA – CA system. (excl = 3000, bin = 0.002, *r_mim_* = 0.13, *r_max_* = 0.2).**


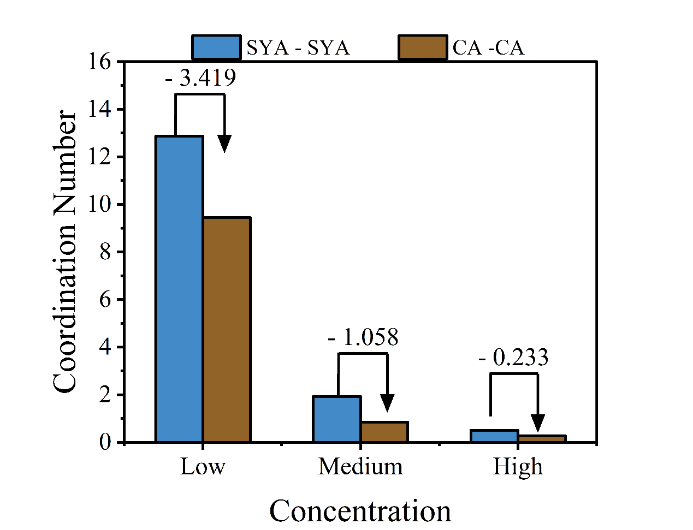


**Figure S10.** **Coordination numbers between monophenols after equilibrium self-assembly in binary mixture systems with different concentrations.**


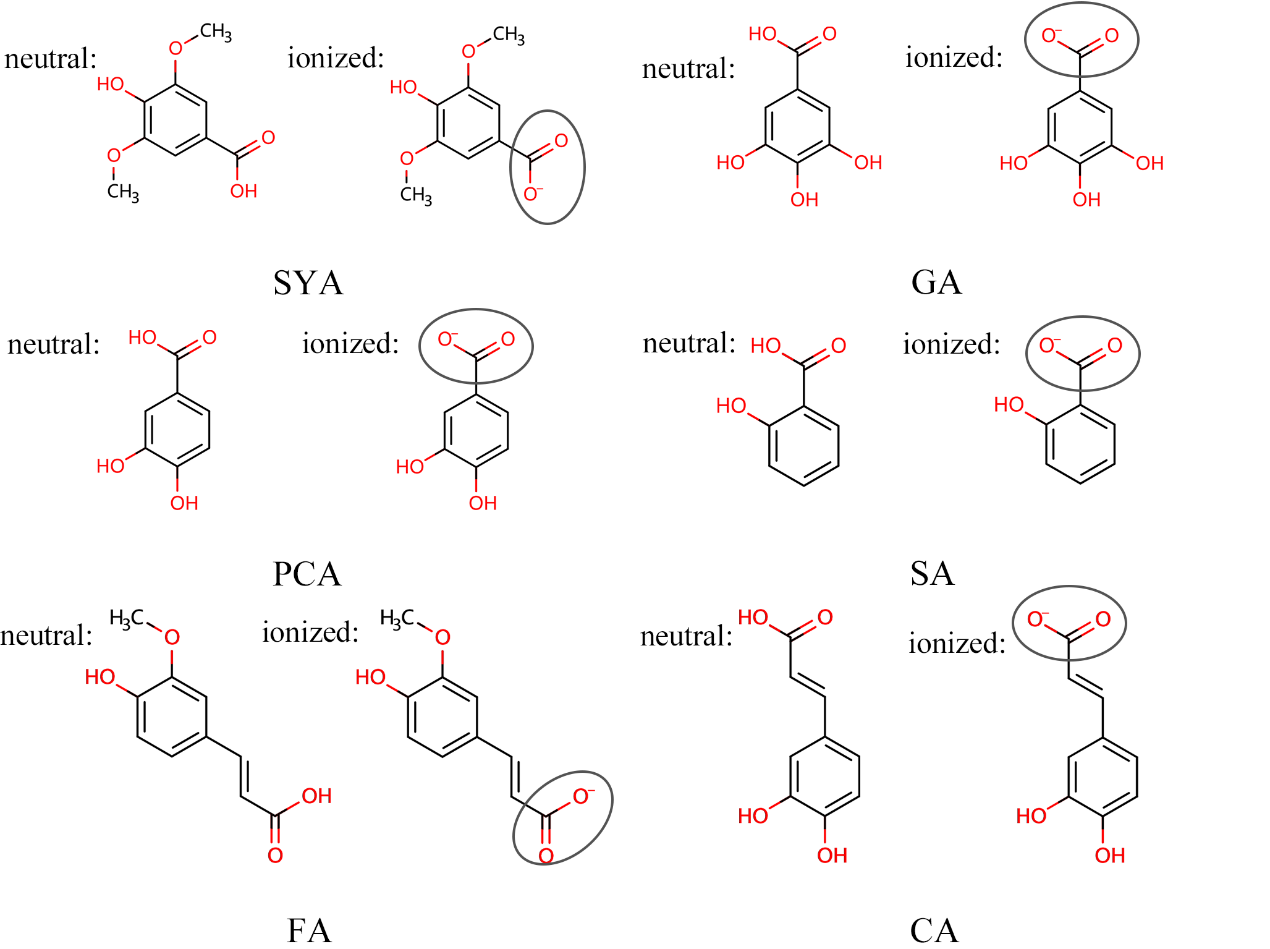


**Figure S11. Chemical structures of various ionization states of Monophenols.**

**References**

[1] Vickers, R., Weigand, T. M., Miller, C. T., & Coronell, O., Molecular methods for assessing the morphology, topology, and performance of polyamide membranes, 2022, J. Membr. Sci., 644, 120110, <https://doi.org/10.1016/j.memsci.2021.120110>

[2] Zhang, X. J., Cahill, D. G., Coronell, O., & Mariñas, B. J., Absorption of water in the active layer of reverse osmosis membranes, 2009, J. Membr. Sci., 331, 143, <https://doi.org/10.1016/j.memsci.2009.01.027>

[3] Wei, T., Zhang, L., Zhao, H. Y., et al., Aromatic Polyamide Reverse-Osmosis Membrane: An Atomistic Molecular Dynamics Simulation, 2016, J. Phys. Chem. B, 120, 10311, <https://doi.org/10.1021/acs.jpcb.6b06560>

[4] Misdan, N., Lau, W. J., Ismail, A. F., Matsuura, T., & Rana, D., Study on the thin film composite poly(piperazine-amide) nanofiltration membrane: Impacts of physicochemical properties of substrate on interfacial polymerization formation, 2014, Desalination, 344, 198, <https://doi.org/10.1016/j.desal.2014.03.036>

[5] Yuan, B. B., Jiang, C., Li, P. F., et al., Ultrathin Polyamide Membrane with Decreased Porosity Designed for Outstanding Water-Softening Performance and Superior Antifouling Properties, 2018, ACS Appl. Mater. Interfaces, 10, 43057, <https://doi.org/10.1021/acsami.8b15883>

[6] Xiang, J., Xie, Z. L., Hoang, M., & Zhang, K. S., Effect of amine salt surfactants on the performance of thin film composite poly(piperazine-amide) nanofiltration membranes, 2013, Desalination, 315, 156, <https://doi.org/10.1016/j.desal.2012.10.038>

[7] Ridgway, H. F., Orbell, J., & Gray, S., Molecular simulations of polyamide membrane materials used in desalination and water reuse applications: Recent developments and future prospects, 2017, J. Membr. Sci., 524, 436, <https://doi.org/10.1016/j.memsci.2016.11.061>

[8] Kolev, V., & Freger, V., Hydration, porosity and water dynamics in the polyamide layer of reverse osmosis membranes: A molecular dynamics study, 2014, Polymer, 55, 1420, <https://doi.org/10.1016/j.polymer.2013.12.045>

[9] Shen, M., Keten, S., & Lueptow, R. M., Dynamics of water and solute transport in polymeric reverse osmosis membranes via molecular dynamics simulations, 2016, J. Membr. Sci., 506, 95, <https://doi.org/10.1016/j.memsci.2016.01.051>

[10] Wang, T. L., Wang, H. L., Ren, L. H., et al., Selective ion permeation enables reduced combined gypsum-organic fouling of loose nanofiltration membranes, 2024, Desalination, 580, <https://doi.org/10.1016/j.desal.2024.117567>

[11] Shen, M., Keten, S., & Lueptow, R. M., Rejection mechanisms for contaminants in polyamide reverse osmosis membranes, 2016, J. Membr. Sci., 509, 36, <https://doi.org/10.1016/j.memsci.2016.02.043>

[12] Gai, J. G., Gong, X. L., Kang, W. L., Zhang, X., & Wang, W. W., Key factors influencing water diffusion in aromatic PA membrane: Hydrates, nanochannels and functional groups, 2014, Desalination, 333, 52, <https://doi.org/10.1016/j.desal.2013.11.028>

[13] Harder, E., Walters, D. E., Bodnar, Y. D., Faibish, R. S., & Roux, B., Molecular Dynamics Study of a Polymeric Reverse Osmosis Membrane, 2009, J. Phys. Chem. B, 113, 10177, <https://doi.org/10.1021/jp902715f>

[14] Liu, S. W., Ganti-Agrawal, S., Keten, S., & Lueptow, R. M., Molecular insights into charged nanofiltration membranes: Structure, water transport, and water diffusion, 2022, J. Membr. Sci., 644, <https://doi.org/10.1016/j.memsci.2021.120057>
